# Prediction of Dhurrin Metabolism by Transcriptome and Metabolome Analyses in *Sorghum*

**DOI:** 10.3390/plants9101390

**Published:** 2020-10-19

**Authors:** Sang Chul Choi, Yong Suk Chung, Yun Gyeong Lee, Yuna Kang, Yun Ji Park, Sang Un Park, Changsoo Kim

**Affiliations:** 1 Department of Crop Science, College of Agriculture and Life Sciences, Chungnam National University, Daejeon 34134, Korea; choisc.wiseman@gmail.com (S.C.C.); yschung@jujunu.ac.kr (Y.S.C.); yglee.gt@gmail.com (Y.G.L.); dkwl3120@cnu.ac.kr (Y.K.); yunji0825@hanmail.net (Y.J.P.); supark@cnu.ac.kr (S.U.P.); 2 Department of Plant Resources and Environment, College of Applied Life Sciences, Jeju National University, Jeju 63243, Korea; 3 Department of Smart Agriculture Systems, College of Agriculture and Life Sciences, Chungnam National University, Daejeon 34134, Korea

**Keywords:** *Sorghum bicolor*, cyanogenic compounds, forage, metabolome, transcriptome

## Abstract

Sorghum (*Sorghum bicolor* (L.)) Moench is an important food for humans and feed for livestock. Sorghum contains dhurrin which can be degraded into toxic hydrogen cyanide. Here, we report the expression patterns of 14 candidate genes related to dhurrin ((S)-4-Hydroxymandelnitrile-*β*-D-glucopyranoside) metabolism and the effects of the gene expression on specific metabolite content in selected sorghum accessions. Dhurrin-related metabolism is vigorous in the early stages of development of sorghum. The dhurrin contents of most accessions tested were in the range of approximately 6–22 μg mg^−1^ fresh leaf tissue throughout growth. The *p*-hydroxybenzaldehyde (pHB) contents were high at seedling stages, but almost nonexistent at adult stages. The contents of *p*-hydroxyphenylacetic acid (pHPAAc) were relatively low throughout growth compared to those of dhurrin or pHB. Generally, the expression of the candidate genes was higher at seedling stage than at other stages and decreased gradually as plants grew. In addition, we identified significant SNPs, and six of them were potentially associated with non-synonymous changes in *CAS1*. Our results may provide the basis for choosing breeding materials to regulate cyanide contents in sorghum varieties to prevent HCN toxicity of livestock or to promote drought tolerance or pathogen resistance.

## 1. Introduction

*Sorghum bicolor* (L.) Moench is one of the most consumable crops in the world. It is an important food resource not only for humans but also livestock and poultry. Sorghum originated from Africa where a wide spectrum of variations is observed [1]. Sorghum is a tropical plant which is well-known for its drought tolerance; therefore, it is amenable to cultivation in regions with low precipitation [2,3]. In addition, it is nutritionally comparable to other crops. In terms of nutrient composition and energy value, sorghum could be an alternative crop to maize for human food and livestock feed and for other uses [2].

Sorghum contains dhurrin ((S)-4-Hydroxymandelnitrile-*β*-D-glucopyranoside), a cyanogenic glycoside, which can be degraded into hydrogen cyanide (HCN). Gaseous HCN is toxic to herbivores and humans. Acute poisoning can lead to sub-lethal symptoms or even death [4,5]. Fortunately, the mature grains only contain a trace amount of dhurrin since the dhurrin accumulated in grains is turned over during grain maturation [6]. Despite the useful properties of sorghum, the cost of forage production or precautions for cyanide poisoning prevention (e.g., silage) may reduce the competitiveness of sorghum.

**Figure 1:**
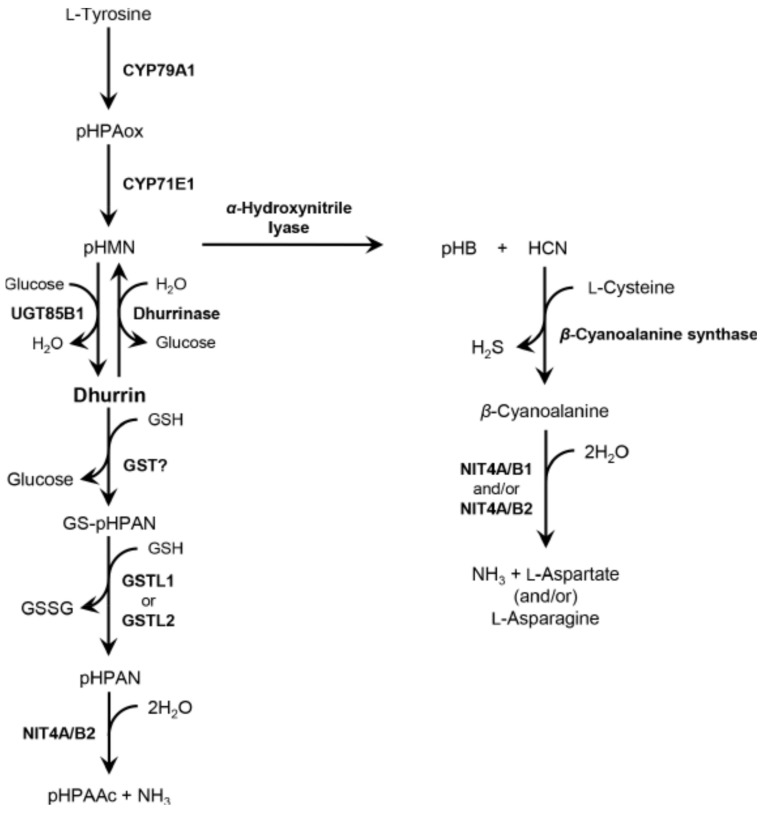
Dhurrin biosynthesis and degradation pathways. Dhurrin, (*S*)-4-Hydroxymandelnitrile-*β*-D-glucopyranoside; pHPAox, (Z)-*p*-hydroxyphenylacetaldehyde oxime; pHMN, *p*-hydroxymandelonitrile; GSH, glutathione; GST, glutathione S-transferase; GSSG, glutathione disulfide; GSTL, glutathione S-transferase-like; GS-pHPAN, glutathionylated *p*-hydroxyphenylacetonitrile; pHPAN, *p*-hydroxyphenylacetonitrile; pHPAAc, *p*-hydroxyphenylacetic acid; NH_3_, ammonia; pHB, *p*-hydroxybenzaldehyde; HCN, hydrogen cyanide; H_2_S, hydrogen sulfide; NIT4, nitrilase 4.

In sorghum, dhurrin is mainly known for acting as a nitrogen storage compound, an osmoprotectant, or a phytoanticipin [7]. Dhurrin is derived from L-tyrosine by three biosynthetic enzymes: two cytochrome P450, CYP79A1 and CYP71E1, and UDP-glycosyltransferase, UGT85B1. CYP79A1 converts L-tyrosine into (Z)-*p*-hydroxyphenylacetaldehyde oxime (pHPAox), and then CYP71E1 catalyzes a conversion of pHPAox to *p*-hydroxymandelonitrile (pHMN). Subsequently, UGT85B1 transfers a glucose molecule to pHMN, producing dhurrin [8,9,10,11]. Dhurrin is degraded through different pathways. The first pathway is a toxic catabolic process (cyanogenesis) in which dhurrin is converted into pHMN by *β*-glucosidase (BGD; dhurrinase), and then *α*-hydroxynitrile lyase (HNL) hydrolyzes pHMN into *p*-hydroxybenzaldehyde (pHB) and HCN [12,13]. Normally, dhurrin and BGD are distributed between different tissues. However, spatial decompartalization of dhurrin and BGD caused by tissue disruption (e.g., herbivore attack or mechanical damage) rapidly generates HCN [14]. The generated HCN undergoes a detoxification process. A typical detoxification pathway is that HCN generated during cyanogenesis reacts with L-cysteine by *β*-cyanoalanine synthase (CAS) to produce *β*-cyanoalanine. Afterward, nitrilase complexes (*Sb*NIT4B2/A or *Sb*NIT4B1/A) produce ammonia (NH_3_) and L-aspartic acid, and/or L-asparagine from *β*-cyanoalanine [15]. The second is a non-toxic catabolic recycling pathway in which unknown enzyme which could be glutathione S-transferase (GST) or BGD catalyze the conversion of dhurrin to *p*-hydroxyphenylacetonitrile (pHPAN) and glutathione (GSH) is needed to detach a glucose from dhurrin [15,16,17]. The glutathionylated-pHPAN (GS-pHPAN) conjugate is converted to pHPAN by GST-like enzymes (GSTL1 or GSTL2) and GSHs [17]. Subsequently, *p*-hydroxyphenylacetic acid (pHPAAc) and NH_3_ are produced from pHPAN by a nitrilase complex (*Sb*NIT4B2/A) [15,18,19]. Until a while ago, the pathway of dhurrin degradation in sorghum was not fully understood but it has been hypothesized that GST could be involved in an alternative pathway that produces pHPAAc with an intermediate pHPAN [7]. Recently, this hypothesis was experimentally demonstrated by the fact that GST enzymes actually convert dhurrin to pHPAN using GSH as a reducing agent, unlike the previous prediction that the production of pHPAAc is due to the action of BGD and unknown factor (Figure 1) [17].

The purpose of our study is to estimate the contents of dhurrin together with its peripheral metabolites using an HPLC system and to profile gene expression patterns associated with dhurrin biosynthesis by quantitative RT-PCR (qRT-PCR) in 17 sorghum accessions provided by various resources, allowing us to investigate the correlations between the metabolite contents and gene expression levels. In addition, we conducted a comparative genomic approach via RNA sequencing (RNAseq) analyses to evaluate the effect of mutation in the relevant genes related to the metabolite content of those sorghum accessions. Our results may provide the basis for choosing breeding materials to regulate cyanide contents in sorghum varieties to prevent cyanide toxicity or to promote drought tolerance or disease resistance, which would be useful for the nutrition of humans and animals.

## 2. Results

### 2.1. Metabolite Analysis at Different Developmental Stages

**Figure 2:**
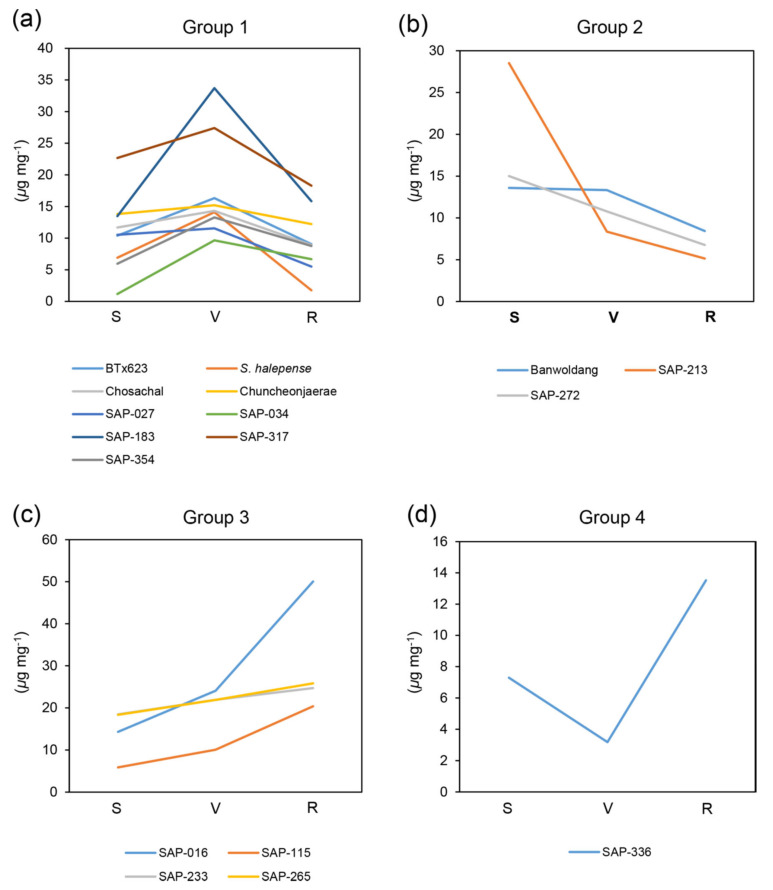
Grouping analysis of dhurrin contents by different developmental stages. The dhurrin contents of all the accessions were classified into four groups according to their patterns. (**a**) Group 1. (**b**) Group 2. (**c**) Group 3. (**d**) Group 4. A separate graph showing the dhurrin contents for each accession is given in the Figure S2. *Y*-axis, amount of dhurrin (μg mg^−1^). *X*-axis, developmental stage. S, seedling stage (Z15/20); V, vegetative stage (Z19/47); R, ripening stage (Z19/87). *n* = 3 or more.

**Figure 3:**
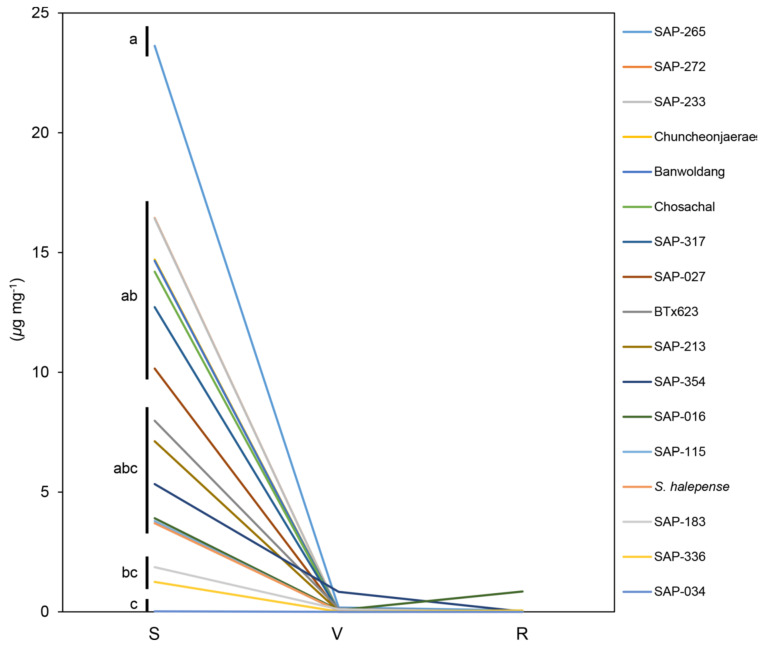
Grouping analysis of pHB contents by different developmental stages. A separate graph showing the pHB contents for each accession is given in the Figure S3. *Y*-axis, amount of pHB (μg mg^−1^). *X*-axis, developmental stage. S, seedling stage (Z15/20); V, vegetative stage (Z19/47); R, ripening stage (Z19/87). Lower-case letters indicate homogeneous subsets for pHB contents only at seedling stage among the accessions (*p* = 0.05). *n* = 3 or more.

**Figure 4:**
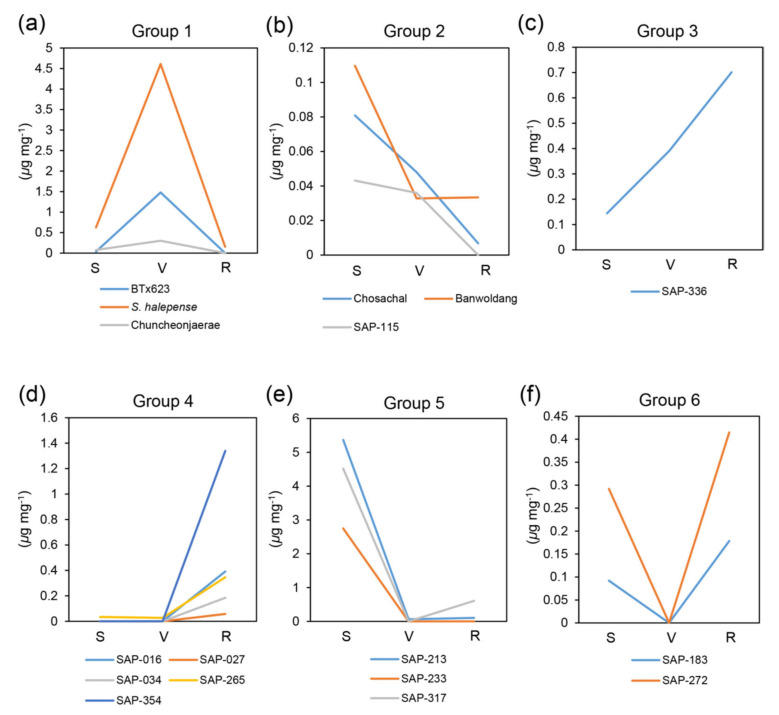
Grouping analysis of pHPAAc contents by different developmental stages. The pHPAAc contents of all the accessions were classified into six groups according to their patterns. (**a**) Group 1. (**b**) Group 2. (**c**) Group 3. (**d**) Group 4. (**e**) Group 5. (**f**) Group 6. A separate graph showing the pHPAAc contents for each accession is given in the Figure S4. *Y*-axis, amount of pHPAAc (μg mg^−1^). *X*-axis, developmental stage. S, seedling stage (Z15/20); V, vegetative stage (Z19/47); R, ripening stage (Z19/87). *n* = 3 or more.

We measured the contents of dhurrin and the intermediates of toxic (pHB) and non-toxic (pHPAAc) pathways at different developmental stages (seedling, vegetative, and ripening stages; see 4. Materials and Methods for definition of the developmental stages) for the 17 sorghum accessions (Figure 2, Figure 3 and Figure 4 and Figures S2–S4, Table S1). Prior to the metabolite analysis, we set experimental conditions to be able to separate those three different metabolites simultaneously under the same conditions in a HPLC system (Figure S1; also see Section 4. Materials and Methods).

The accessions were then classified into the same group according to the pattern of contents for each metabolite by different developmental stages (Figure 2 and Figure 4). However, since the patterns of pHB contents were almost similar in all the accessions, we divided them into subgroups by the homogeneity of the variance analysis based on the pHB content at the seedling stage (Figure 3).

The dhurrin contents of all the accessions were classified into four groups. In group 1, patterns of dhurrin contents increased at vegetative stage compared to seedling stage and decreased at ripening stage, and this pattern appeared most frequently (Figure 2a). Group 2 and group 3 showed gradually decreased or increased patterns for them according to developmental stages, respectively (Figure 2b,c). A pattern of dhurrin contents were significantly decreased at vegetative stage in comparison with at seedling stage and then increased again at ripening stage, and only SAP-336 showed this pattern (Group 4) (Figure 2d).

The dhurrin contents for all the accessions were comparable (approximately ranging from 6 to 22 μg mg^−1^ fresh leaf tissue; Figure S5), except SAP-016, in which the dhurrin content remained at a high concentration at the ripening stage compared to that in other accessions (Figure 2, Figures S2 and S5). Among the accessions having significant levels for the dhurrin contents, at seedling stage, SAP-034 showed lower dhurrin content, but SAP-213 showed higher dhurrin content compared to other stages. In BTx623, *Sorghum halepense*, and SAP-183, the dhurrin contents at the vegetative stage were higher than those at other stages of each accession. During growth, gradually, the dhurrin contents of SAP-115 and -233 increased slightly, but those of SAP-272 decreased. In SAP-336, the dhurrin content was reduced at vegetative stage, but increased at ripening stage compared to that at seedling stage (Figure 2, Figures S2 and S5).

The pHB contents were high at seedling stage, but almost nonexistent or lower at vegetative and ripening stages than seedling stage (Figure 3, Figures S3 and S5). However, the pHB contents showed difference at the seedling stage (Figure 3). Of all the accessions with significant level of the pHB contents at seedling stage, SAP-265 contained the highest pHB content (23.6 μg mg^−1^) at seedling stage (subgroup a), and Chuncheonjaerae, Chosachal, Banwoldang, SAP-027, -233, -272, and -317 showed relatively higher the pHB content (10 μg mg^−1^ or more; subgroup ab) compared to other stages in each accession. The pHB contents of BTx623, *S. halepense*, SAP-016, -115, -213, and -354 showed the range of about 2–10 μg mg^−1^ (subgroup abc). SAP-183 and -336 were lower than 2 μg mg^−1^ (subgroup bc) while SAP-034 contained very low pHB content (<0.02 μg mg^−1^; subgroup c). On the other hand, the pHB content remained higher in SAP-354 at vegetative stage and in SAP-016 at ripening stage compared to those in other accessions (Figure 3, Figures S3 and S5).

The pHPAAc contents showed various patterns for all the accessions. They were classified into six groups. Group 1 showed pHPAAc patterns with increase at vegetative stage and decrease at ripening stage to the similar amount to the contents of seedling stage (Figure 4a). Patterns of gradual decrease and increase for pHPAAc contents belonged to group 2 and group 3, respectively (Figure 4b,c). In group 4, the pHPAAc contents were significantly increased at ripening stage while almost non-detectable at seedling and vegetative stages (Figure 4d). Group 5 had very high contents at seedling stage, but the contents were significantly reduced at adult stages (vegetative and ripening stages). In the group 5, the pHPAAc contents were very high at the seedling stage compared to the other accessions (Figure 4e). In group 6, a pattern of pHPPAc contents were significantly decreased at vegetative stage in comparison with seedling stage and then the contents at ripening stage were elevated higher than those at seedling stage (Figure 4f). Unlike the other groups, groups 2 and 6 showed generally low pHPAAc contents (Figure 4). At vegetative stage, the pHPAAc content was the highest in *S. halepense* (Figure 4, Figures S4 and S5). The pHPAAc contents in SAP-213, SAP-233, and SAP-317 markedly exceeded the whisker range at seedling stage. *S. halepense* and BTx623 had the outlier values at the vegetative stage, and SAP-354 showed the outlier value at the ripening stage (Figure S5).

### 2.2. Identification of Genes Involved in Dhurrin Metabolism

Previous studies have shown that several genes control the pathways of biosynthesis and catabolism of dhurrin. We selected candidate genes which are relevant to putative dhurrin biosynthesis and degradation, as well as already-known genes based on earlier reports (Table S2). Sequences of 14 candidate genes were collected from *S. bicolor* (v3.1.1) database in Phytozome (v12.1; https://www.phytozome.net/) [20]. Among candidate genes, two genes (Sb08g007586 and Sb08g007650) were highly homologous to *Dhr1* (Sb08g007570) with 93.6% and 94.7% amino acid sequence identities, respectively [21]. The description of those genes was “similar to Dhurrinase” in the Phytozome database, and they carry a domain Pfam: Glyco_hydro_1 (PF00232) that Dhr1 and Dhr2 also have (http://smart.embl-heidelberg.de/); therefore, we putatively assigned them as paralogous genes to *Dhr1*, *Dhr3,* and *Dhr4*, respectively (Table S2). Three paralogs of *HNL* (Sb04g036350, Sb05g024440, and Sb05g024460) exist in the sorghum genome. In previous study, Sb04g036350 was identified as a homolog of serine carboxypeptidases (*SbHNL*; GenBank accession no. AJ421152) [13], and the *SbHNL* expression has been also analyzed through transcriptome studies [6,21]. Thus, we selected only Sb04g036350 and designated it as *HNL1*. Expression of *CAS* in the developing grains and its tissue distribution in leaves were only investigated in the past [6,22], and is seems to be a single copy gene based on our similarity search. Therefore, this gene was named as *CAS1* and included in the set of candidate genes. In recent, GSTLs have been revealed to be involved in the dhurrin recycling pathway; thus, we also selected two *GSTL* genes: *GSTL1* (Sb02g043250) and *GSTL2* (Sb09g002800) (Table S2) [17].

### 2.3. Quantitative RT-PCR Analysis of Candidate Genes

To determine the expression of genes involved in the biosynthesis and degradation pathways of dhurrin, transcript levels of the relevant genes were examined by qRT-PCR according to developmental stages (Figure 5 and Figures S6–S21).

Genes involved in dhurrin biosynthesis were expressed in two different patterns. *CYP79A1*, *CYP71E1*, and *UGT85B1* expressed highly at seedling stage while those expressions decreased significantly or were not detectable at adult stages (Figure 5 and Figures S6–S21). Exceptionally, in SAP-115 and -233 accessions, transcript levels of *CYP79A1* remained at more than half of seedling stage levels at vegetative stage and decreased gradually during growth. Transcript levels of *CYP71E1* and *UGT85B1* rather increased at the vegetative stage and then decreased drastically at ripening stage compared to those at seedling stage (Figures S13 and S16).

*Dhr1*, *Dhr2*, and *Dhr4* were highly expressed at seedling stage, while those expressions were almost non-detectable at adult stages. The relative transcript level of *Dhr2* was much higher than that of other *Dhr* genes (Figure 5 and Figures S6–S21). The expression pattern of *Dhr3* was similar to other *Dhr* genes at seedling stage whereas *Dhr3* expressions decreased significantly at the vegetative stage, and then elevated slightly at ripening stage in some accessions (SAP-183, -213, -272, -317, -336, and -354). In SAP-183, *Dhr3* expression was higher at ripening stage than at seedling stage (Figures S14, S15 and S18–S21).

Expressions of *NIT4A* and *NIT4B2* showed very similar patterns for the entirety of the accessions. Generally, expressions were high at seedling stage, and then reduced gradually during growth. On the other hand, *NIT4B1* expressions were in a relatively constant range or showed the opposite expression patterns compared to expressions of *NIT4A* and *NIT4B2* (Figure 5 and Figures S6–S21). In SAP-016, *NIT4B1* expressions declined gradually during growth with high relative expression level (Figure S10).

*HNL1* expressed at the highest level at seedling stage, and after that stage, expression patterns of *HNL1* appeared as different ways. First, during growth its expression decreased greatly at vegetative stage and then remained low level at ripening stage. Second, its expression reduced steadily during growth. Third, its expression was maintained at a similar level at the vegetative stage compared to the seedling stage and then decreased significantly at the ripening stage. Fourth, its expression was maintained at a relatively high level throughout the growth (Figure 5 and Figures S6–S21).

*CAS1* was expressed in a manner similar to *HNL1*. *CAS1* expression was higher at seedling stage than other stages and decreased gradually during growth (Figure 5 and Figures S6–S21). However, in some accessions (BTx623, Banwoldang, SAP-027, -034, and -183), transcript levels of *CAS1* were reduced at the vegetative stage but elevated at ripening stage (Figure 5, Figures S9, S11, S12 and S14). Exceptionally, the expression of *CAS1* in *S. halepense* was maintained at a very high level throughout the growth (Figure S6).

Expressions of *GSTL1* were increased (SAP-115 and -354; Figures S13 and S21) or decreased (SAP-183, -233, -265, 272-, and -317; Figures S14 and S16–S19) during growth. Some accessions (SAP-034 and -336) showed a decreasing expression pattern at the ripening stage after an increase at vegetative stage (Figures S12 and S20). Remaining accessions showed no significant difference in *GSTL1* expression pattern throughout all the growth stages. Expressions of *GSTL2* were divided into two patterns. One was little or no change in the expression pattern of *GSTL2*, and the other showed gradually increased expression during growth (Figure 5 and Figures S6–S21).

### 2.4. Correlation of Metabolite Contents and Gene Expression Levels

To investigate correlation between the metabolite contents and the gene expressions involved in dhurrin metabolism, Pearson’s correlation coefficients were calculated based on their relevance (Table 1). As a result, there were statistical significances for the expressions of the almost genes on the pathway of dhurrin biosynthesis, cyanogenesis, and detoxification. The expression levels of *NIT4A*, *NIT4B2*, and *CAS1* showed strong positive correlation with each other (*p* < 0.01). However, the expressions of *UGT85B1* were closely correlated with the biosynthesis genes. Interestingly, the *GSTL2* expressions showed significant but negative correlation with the expressions of some genes, and no correlation was observed in the expressions of *NIT4B1* with any metabolite contents or gene expressions (Table 1).

### 2.5. SNP Analysis through RNAseq

**Figure 5:**
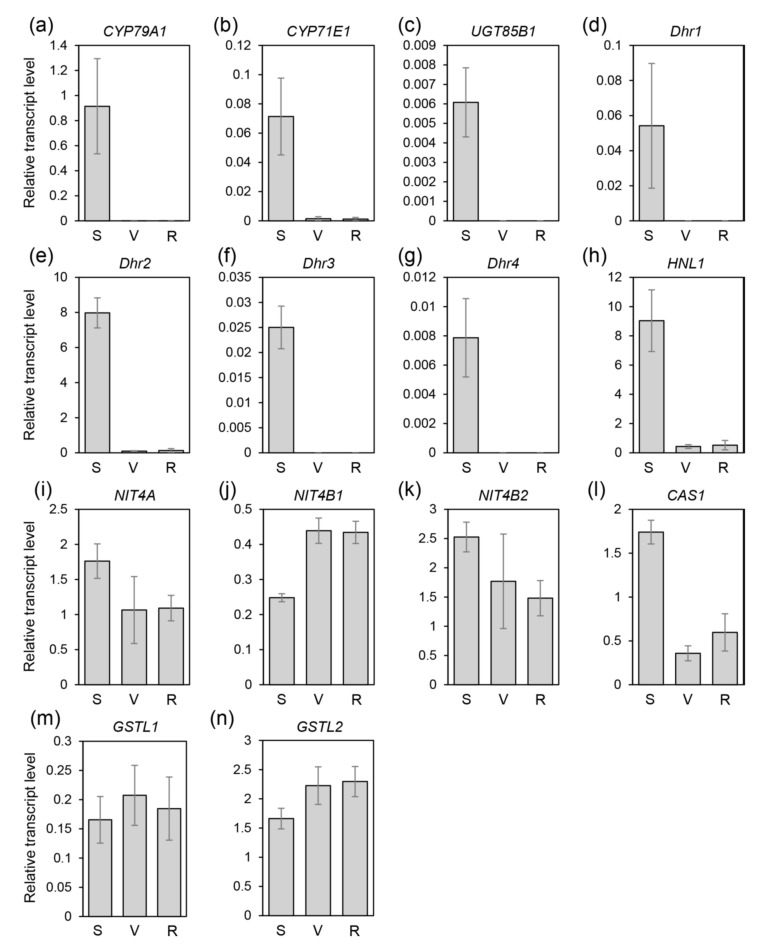
Expression patterns of candidate genes for dhurrin metabolism according to different developmental stages in BTx623. Quantitative real-time RT-PCR analyses of (**a**) *CYP79A1*, (**b**) *CYP71E1*, (**c**) *UGT85B1*, (**d**) *Dhr1*, (**e**) *Dhr2*, (**f**) *Dhr3*, (**g**) *Dhr4*, (**h**) *HNL1*, (**i**) *NIT4A*, (**j**) *NIT4B1*, (**k**) *NIT4B2*, (**l**) *CAS1*, (**m**) *GSTL1*, (**n**) *GSTL2*. *Y*-axis, transcript level relative to sorghum *PP2A* expression. *X*-axis, developmental stage. S, seedling stage (Z15/20); V, vegetative stage (Z19/47); R, ripening stage (Z19/87). Values are shown as means. Error bars indicate standard deviation. *n* = 3 or more.

We selected four accessions, BTx623 (reference), *S. halepense* (wild species), SAP-336 (unique metabolomics profile for dhurrin and pHPAAc contents compared to others), and -354 (the highest pHPAAc at the ripening stages) out of tested accessions and performed RNAseq. A total of 72 Gbp sequence reads were generated using Illumina HiSeq2500 platform. The reads were mapped to the *S. bicolor* (v3.1.1) reference assembly at an average coverage of 19–49 (Table S3). Using these contigs, we obtained expression profiles of 12 candidate genes related to dhurrin biosynthesis and catabolism represented as fragments per kilobase of exon per million reads mapped (FPKM) value (Figures S22–S25). As a result, the overall patterns were consistent with the qRT-PCR results (Figure 5 and Figures S6–S21), indicating that the qRT-PCR analysis was validated by FPKM analysis obtained from RNAseq. Therefore, we used the RNAseq data for further study.

Next, to obtain SNPs associated with the genes of dhurrin biosynthesis and catabolism from the representative accessions, raw RNA data of each sample at three developmental stages were used. We obtained a total of 2701 SNPs from 12 sorghum samples (Table S4). All the filtered SNPs were annotated and categorized into four impact effects using SnpEff, a SNP annotation tool [23]. Among them, 15 SNPs with high impacts were finally selected by removing overlapped SNPs across accessions However, only two genes, *NIT4B2* and *CAS1*, were covered by the SNP analysis (Table 2 and Table S4; see Section 4. Materials and Methods for details). The result showed that one SNP located in the fourth intron of *NIT4B2* gene and 14 SNPs distributed in an upstream region of the coding sequence of *CAS1*. Out of the 14 SNPs, two SNPs were located at intron, and six SNPs were at 5′ UTR. Six SNPs were potentially associated with non-synonymous changes in *CAS1*. Based on the results of significant SNP markers, we reconstituted the nucleotide sequence and checked change of amino acid sequence by non-synonymous substitutions. SNPs located at the first exon of *CAS1* could cause a pre-mature stop codon at the second exon by a frameshift mutation in *S. halepense* (Figures S6 and S26; Table 2).

## 3. Discussion

### 3.1. Correlation of Metabolite Contents and Expression Profiles

We showed that the contents of pHB were the highest at the seedling stage for all the accessions. In addition, all the accessions showed high levels of expressions for the dhurrin biosynthetic and *Dhr* genes tested at seedling stages. Taken together, these results indicate that dhurrin-related metabolism is vigorous in the early stages of development (Figure 2, Figure 3 and Figure 5, and Figures S6–S21) and are consistent with previous studies [7,24,25]. In addition, there were generally significant correlations between the patterns of the pHB content and the expression levels of genes involved in dhurrin biosynthesis, catabolism through BGD, and detoxification. The expression patterns of *GSTL1* were significantly correlated with those of *NIT4A*, *NIT4B2*, and *CAS1* (*p* < 0.05). It is presumed that GSTL1 is more closely associated with the NIT4A/B2 complex in the dhurrin recycling pathway (Table 1). However, we could not find any clear correlations between the patterns of metabolite contents and expressions of related genes across all the accessions. This is probably due to the complicated interactions that occur in catabolism with two or more pathways after dhurrin biosynthesis.

However, our results suggest some possibilities. SAP-016 showed a higher level of dhurrin than other accessions (Figure 2 and Figure S2). The specificity of this accession is that not only the expression patterns of *NIT4A*, *NIT4B1*, and *NIT4B2* were similar to those of *HNL1* and *CAS1*, but also the expression levels of *GSTL1* and *GSTL2* increased at the ripening stage compared to the previous stages (Figure S10). Since the genes involved in the degradation and detoxification of dhurrin might be reduced in their expressions, the dhurrin content in SAP-016 seemed to be gradually increased. Moreover, the pHPAAc content was elevated significantly at the ripening stage. This is consistent with the expected results of increased expression of *GSTL* genes. If the expression patterns of these genes are similar in other accessions, then the dhurrin content of the accession would be expected to be high. Another noticeable point is that the patterns of the gene expression of *NIT4*s and *GSTL*s and the contents of dhurrin and pHPAAc were similar according to developmental stages, and the expression of *CAS1* is consistently high regardless of developmental stage in *S. halepense* (Figure 2 and Figure 4 and Figure S6). Previous studies reported that HCN concentrations were high throughout the growth and the cyanide content was the highest in the middle of development in *S. halepense* [26]. In *S. halepense*, detoxification is likely to be more active than recycling for dhurrin metabolism. Although no definitive correlation has been established in our study, further studies for more sorghum accessions may reveal a correlation between the contents of dhurrin and its derivatives, and the expression patterns of the genes related to dhurrin metabolism.

### 3.2. Function of Dhurrinases

Dhr proteins play a role in removing glucose from dhurrin, and four paralogs of *Dhr* genes are found in the sorghum genome. *Dhr1* and *Dhr2* have already been studied through various approaches [6,21,27,28,29]. *Dhr3* and *Dhr4* are expected to encode a putative Dhr, but there has been little research on its expression or function. The researchers did not annotate them as pseudogenes, and transcriptome analysis confirmed the expressions of these genes, although not as high as *Dhr1* and *Dhr2* [21]. Moreover, the study using diverse sorghum conversion lines showed the possibility that the *Dhr*-like gene, not previously involved in the dhurrin metabolic pathway, might be implicated in the dhurrin metabolism [30]. Our results are consistent with the previous study regarding the expressions of Dhr genes, and we confirmed that the expression level of *Dhr2* is higher than that of other *Dhr* genes (Figure 5 and Figures S6–S21). Dhr1 and Dhr2 take dhurrin as physiological substrates, and Dhr2 showed enzymatic activity against 4-Methylumbelliferyl-*β*-D-glucuronide (4MUG) as a synthetic substrate. Dhr2 protein stabilizes its substrate by key amino acid residues in a binding pocket, thereby having a broader substrate specificity compared to Dhr1 [31]. However, previous studies did not reveal which protein, Dhr1 or Dhr2, degrades dhurrin through its respective degradation pathway. Presumably, differences in amino acid sequence between Dhr1 and Dhr2 (showing 70% amino acid sequence identity with each other) determine substrate specificity.

During the process of dhurrin degradation, pHPAN is probably produced by a sequential process; the removal of glucose and dehydroxylation. It is expected that unknown protein co-factors may participate in this conversion process, because Dhr does not have dehydroxylation activity. A previous study reported that maize BGD (Glu) binds to BGD aggregating factor (BGAF) to form an insoluble aggregate, and BGAF was identified as a jacalin-related lectin (JRL) [32]. Sorghum lectin (SL) was identified as a homolog of JRL in sorghum, and SL did not interact to Dhr1 differing from BGAF against Glu [33]. The deletion of dirigent domain in BGAF and SL led to an abolishment of specificity to sugar [34]. Through these results, determination of substrate specificity by protein co-factor is a reasonable explanation, even though Dhr2 has broad substrate specificity.

Based on these hypotheses, we can consider the following possibilities. First, *Dhr* genes may be functionally differentiated by spatial compartmentalization resulting from differential tissue expression. Dhr3 and Dhr4 could show differential function, since gene expression pattern or enzymatic activity of Dhr1 and Dhr2 are different in different tissues [27,28]. Second, Dhr proteins may cause substrate specificity or functional change by forming hetero multimers. Dhr1 and Dhr2 form a tetramer, and Dhr1 has at least three isomeric forms [27]. Therefore, we cannot rule out the possibility of heteromer formation among Dhr proteins. During development, expression of all *Dhr* genes is highest at the seedling stage (Figure 5 and Figures S6–S21), supporting the possibility of Dhr protein–protein interactions. The identification of glucosidase or unknown protein that convert dhurrin to pHPAN is a key point to understand dhurrin recycling, recent study has revealed that GSTLs converts GS-pHPAN conjugate into pHPAN in the process of converting dhurrin to pHPAN [17]. However, an enzyme that makes dhurrin a GS-pHPAN conjugate is not yet clear. If dhurrin can be converted to GS-pHPAN by BGD, we consider the chance that Dhr3 is involved in the recycling pathway, because the expression of *Dhr3* remains at a fairly high level at the adult stage compared to the seedling stage for several sorghum accessions (Figures S14, S15 and S18–S21). The study of gene knockout through the generation of transgenic sorghum plants for *Dhr* will help to understand the function of Dhr in the degradation of dhurrin, which is planned for our future research.

### 3.3. Complex Dhurrin Metabolism

An earlier study proposed that a recycling pathway for cyanogenic glucosides without releasing toxic HCN. This study identified *p*-glucosyloxyphenylacetic acid (pGPAAc), dhurrin diglucosides, caffeoyl-dhurrin, and caffeoyl-dhurrin acid as well as dhurrin amide and dhurrin acid, thereby suggesting that dhurrin can be converted to a variety of glucosylated compounds, and cyanide functional group of dhurrin is able to be substituted into carboxyl or amide group [19]. More recently, pGPAAc was detected in sorghum seedling, and the function of *GSTL* genes to convert dhurrin to pHPAN is connected to the establishment of the recycling pathway [17]. The relevant genes for dhurrin metabolism are limited, and the interconversion of dhurrin and its derivatives seems to be complicated. However, our study was conducted mainly on the analysis of the metabolite contents which can be stably confirmed with the expression of well-known genes in dhurrin metabolism. Therefore, in future studies, our results together with previous transcriptome analysis of dhurrin metabolism [6] will be clues and help in the identification and characterization of enzymes involved in the formation of these dhurrin derivatives, providing advanced reason why dhurrin is required in sorghum.

### 3.4. The Effect of Gene Expressions on HCN Detoxification

In our study, expressions of *HNL1* and *CAS1* showed the same patterns in most cases, but with slight variations (Figure 5 and Figures S6–S21). The reasons may be interpreted in two ways. First, high pH value (higher than pH 6) leads to the spontaneous dissociation of pHMN to pHB and HCN, but the support of HNL1 is required for that reaction to occur at lower pH values [5]. It would be difficult to measure the ratio between the amount being spontaneously degraded and the amount decomposed by HNL1. In addition, there may be differences in in vivo conditions such as intracellular pH depending on the accession. Second, the generation of HCN is not only due to dhurrin catabolism but also to ethylene biosynthesis [35]. Based on the evidence, the similar expression patterns of *HNL1* and *CAS1* are not due to their working simultaneously but because CAS1 may be activated by HCN which is produced by HNL1. In other words, the activation of CAS1 may be dependent upon the concentration of HCN in plant tissues.

### 3.5. Functional Differentiation of NIT4 Enzymes

The action of nitrilase heterocomplex is required for dhurrin catabolism and detoxification processes [15]. We showed the similar expression patterns that exist between *NIT4A* and *NIT4B2* (Figure 5 and Figures S6–S21), supporting the formation of a complex between NIT4A and NIT4B2 enzymes in sorghum. The NIT4A/B2 complex may act preferentially toward the degradation of pHPAN while NIT4A/B1 may function predominantly toward in *β*-cyanoalanine degradation [15]. However, the expression patterns of NIT4B1 were different from those of NIT4A and NIT4B2 in most accessions, presumably due to functional differentiation.

In a previous study, during grain maturation, the expression of *ACC oxidase* was shown to correlate with the expression of *CAS1* and *NIT4B1*, suggesting that CAS1 and NIT4B1 are primarily involved in detoxification of HCN produced during ethylene production [6]. In our results, *NIT4B1* had no significant correlation with the metabolite contents or the expressions of other genes in its expression, and the similar expression patterns of *CAS1* and *NIT4B1* appeared only in SAP-016 (Table 1 and Figure S10). A relationship between ethylene and the expression patterns of the two genes could not be clarified, but persistent accumulation of dhurrin may be involved in common expression patterns of genes related to catabolism and detoxification and high expression level of *NIT4B1*.

### 3.6. Identification of SNPs in Dhurrin Degradation

In the SNP analysis, SNPs in the *CAS1* gene showed that detoxification may be involved in major differences in the dhurrin biosynthesis and degradation pathways between domesticated sorghum and wild relative species. *S. halepense*, also known as johnsongrass, is a tetraploid wild species which was formed by natural hybridization of ancient genome progenitors (ca. 1–2 million years ago (MYA) that was after the divergence between *S. bicolor* and *S. propinquum*) but its subgenomes resemble those two possible genome progenitors [36]. In our results, unlike other accessions, the expressions of *CAS1* in *S. halepense* remained high throughout the growth period (Figure S6). However, if the changes by all the SNPs analyzed have occurred, the protein may not be functional since the deduced protein from the transcript of *CAS1* in *S. halepense* is truncated due to pre-mature stop codon. It is possible that upstream of detoxification pathway recognizes HCN and regulates downstream signaling as feedback, inducing continuous *CAS1* expressions despite its inability to normally detoxify HCN. In addition, 5′UTR contains various regulatory elements and controls translation initiation; therefore, transcription factors or other elements may play a role in translation initiation and control of gene expression through the 5′UTR [37]. In contrast, if there is a partial effect of SNPs on the *CAS1* sequence of *S. halepense* compared to BTx623, non-synonymous substitution of amino acid may have affected the *CAS1* expression. Presumably, the high expression level of *CAS1* at the adult stage of *S. halepense* is regarded to be due to this reason (Figures S6 and S26). This needs to be further investigated in our future research, so that we can get some clues to use *S. halepense* as a useful genetic resource for sorghum breeding programs.

### 3.7. Perspectives of Sorghum Materials and Breeding

Due to the adverse weather effects that have occurred in the world in recent decades, many regions are suffering from natural disasters such as drought. This emphasizes the need to select crops that can respond to the environmental changes. In the past, the United States had developed a sorghum production system by introducing novel genetic variations of diverse germplasms into existing cultivars through Sorghum Conversion Program (SCP) by USDA and Texas A&M University [38]. The SCP lines contain various genetic variations of tropical sorghum [39]. Previous studies have shown that these SCP lines had evaluated for phenotypic variation such as plant height, flowering time, and panicle length through association mapping [40,41]. Recently, SNP analysis using various SCP lines have shown that the content of dhurrin is high in accessions where both biosynthesis and catabolism of dhurrin are activated [30]. There is, of course, a need for further research on whether the related accessions with high dhurrin content are indeed resistant to drought-like stresses. In this regard, SAP lines are an excellent material for these studies and provide good genetic materials to breeders. In this study, we investigated the contents of pHB and pHPAAc generated from the catabolism pathway of dhurrin in various accessions. For example, SAP-016 with high dhurrin content during growth (Figure 2 and Figure S2) would be useful materials for the identification of the function of dhurrin and the line breeding. Unfortunately, we need more evidence and empirical data to correlate metabolite contents to gene expressions across all the accessions. Nonetheless, we provided some background information for the contents of dhurrin, pHB, and pHPAAc according to different developmental stages and a variety of accessions and attempted to determine whether the contents correlated with the expression of related genes. We hope that a series of our planned research will contribute to the selection of breeding materials for controlling cyanide compounds in sorghum.

## 4. Materials and Methods

### 4.1. Plant Materials and Growth Condition

We collected the 17 sorghum accessions: Fourteen accessions of SAP [40] were obtained from the University of Georgia (in Athens, GA, USA) while the reference accession BTx623 and three local varieties (Chosachal, Chuncheonjaerae, and Banwoldang) were obtained from National Agrobiodiversity Center of Rural Development Administration in Korea. One wild accession (*S. halepense*) was obtained from the Wild Plant Resources Seed Bank of Korea University in Korea. Other than *S. halepense*, all the accessions used in this study are *S. bicolor* (Table S1).

Sorghum plants were grown in a plastic greenhouse located on the experimental field of Chungnam National University (36°22′7.57″ N, 127°21′14.92″ E). The experiment was conducted from May 2016 to August 2016 under natural conditions. After treatment of disinfection reagent, seeds were sown in 32-well plastic tray using bed soil. After 26 days after sowing (DAS), seedlings were transplanted in 25 cm-diameter plastic pots. We used the Zadoks growth scale to describe the exact developmental stage for when leaf samples were harvested: seedling stage (Z15/20; 26 DAS), vegetative stage (Z19/47), and ripening stage (Z19/87) [42]. Before transplanting, a part of seedlings was harvested as seedling samples. To ensure minimum dry weight (more than 200 mg), above-aerial parts of the seedlings were sampled. At proper developmental stages in each accession, vegetative and ripening samples were harvested individually from leaf blades since the accessions showed different flowering time and leaf senescence. Leaf blades from the uppermost first to the fourth were pooled. The samples were frozen in liquid nitrogen and then stored in −80 °C until use.

### 4.2. Metabolite Extraction

The frozen samples were put into a plastic zipper bag with a number of 5 mm diameter holes and freeze-dried (IlShin, Yangju, Republic of Korea) at −80 °C for 72 h. The freeze-dried samples were ground into a fine powder in liquid nitrogen using sea sand (20–30 mesh), and mortar and pestle. Previous report was referred to extract metabolites [43]. Based on the weight of the sample itself, 200 mg of each sample was transferred into 15 mL tube and then 6 mL of 80% methanol was added. The sample was vortexed well and sonicated for 30 min at room temperature using ultrasonic water bath (Emerson, St. Louis, MO, USA) with vortexing every 10 min. After sonication, the sample was centrifuged at 12,000× *g* for 10 min at 4 °C. The supernatant was filtered through 0.45 μm PVDF filter (Futecs, Daejeon, Republic of Korea). The filtered extract was analyzed promptly by a HPLC system.

### 4.3. HPLC Analysis

The pHB is produced in equimolar amounts to HCN during dhurrin degradation [44]. Therefore, measuring the amount of HCN is replaceable by measuring pHB content in sorghum plants. The conventional method is to estimate dhurrin content by measuring the total amount of HCN potential using specific degradative BGD (e.g., almond emulsin). However, this method is not suitable for measuring intact HCN produced naturally by catabolism in vivo; thereby, we estimated the contents of dhurrin and pHB directly to check pHB content in vivo using a HPLC system. In the same manner, pHPAAc generated through an alternative detoxification pathway was measured directly using a HPLC system.

The HPLC analysis was performed as previously described with minor modifications [45]. The standard chemicals of dhurrin (CAS no. 499-20-7), *p*-hydroxybenzaldehyde (CAS no. 123-08-0), and *p*-hydroxyphenylacetic acid (CAS no. 156-38-7) were obtained from Sigma-Aldrich. HPLC analysis was performed using NCS-4000 HPLC system (Futecs, Daejeon, Republic of Korea) using a C18 column (250 mm × 4.6 mm, 4 μm; Phenomenex, Torrance, CA, USA). The flow rate was maintained at 1.0 mL min^−1^ and the column was maintained at 30 °C. The injection volume was 20 μL. The mobile phases consisted of methanol (A) and 2% acetic acid in water (B), and a gradient elution protocol was used. Samples were eluted with the following gradient: 0 min, 80% A/20% B; 1 min, 80% A/20% B; 21 min, 55% A/45% B; 24 min, 55% A/45% B; 25 min, 30% A/70% B, 29 min, 30% A/70% B; 30 min, 80% A/20% B; and 34 min, 80% A/20% B. The ultraviolet (UV) detection wavelength was 280 nm for the metabolites. The concentration of each compound was determined using a standard curve.

### 4.4. RNA Extraction and cDNA Synthesis

The samples were ground into fine powder in liquid nitrogen using sea sand (20–30 mesh), and mortar and pestle. Total RNA was extracted using RNAiso plus (Takara, Tokyo, Japan) and treated with DNase I (Promega, Madison, WI, USA) under manufacturer’s instructions. The quality and quantity of the RNA were measured using a PDA UV-Vis Spectrophotometer (Scinco, Seoul, Republic of Korea).

First-strand cDNA was synthesized with 2 µg of total RNA using M-MLV reverse transcriptase (Promega, Madison, WI, USA) under manufacturer’s instructions. Briefly, 100 pmol of oligo (dT)18 was annealed to 2 µg of total RNA at 70 °C for 10 min and cooled down immediately on ice. Afterward, M-MLV reverse transcriptase (Promega, Madison, WI, USA), 5X M-MLV reverse transcriptase buffer (Promega, Madison, WI, USA), dNTP (BioFact, Daejeon, South Korea), and rRNasin (Promega, Madison, WI, USA) were added to the reaction. The reaction was incubated at 42 °C for 1 h and inactivated at 95 °C for 5 min. The synthesized cDNA was diluted 6-fold with distilled water.

### 4.5. Quantitative RT-PCR Analysis

For qRT-PCR, gene-specific primers were designed using Primer3web version 4.0.0 (http://bioinfo.ut.ee/primer3/) or manually. The cDNA was used for expression analysis in a CFX96 Real-Time System (Bio-rad, Hercules, CA, USA). To ensure primer specificity, data were analyzed when the melting curve showed a single peak. The amplification efficiency of primers was verified using Quality Control provided in Bio-Rad CFX Manager 3.1 software (Bio-rad, Hercules, CA, USA), and primers having out of the default were discarded. The reactions were performed in a 20 µL containing 10 µL of SYBR Master Mix (SmartGENE, Daejeon, Republic of Korea), 5 pmol of forward primer, 5 pmol reverse primer, and 2 µL of diluted cDNA. Three-step amplification protocol were conducted: 10 min at 95 °C followed by 45 cycles of 15 s at 95 °C, 30 s at 57 °C, and 30 s at 72 °C). The *S. bicolor Serine/threonine-protein phosphatase 2A* (*PP2A*, GenBank accession no. XM_002453490) [46] served as a reference to normalize the cDNA quantity. The ΔΔCt method was used to calculate gene expression levels. Primers for analyzing gene expression are provided in the Table S5.

### 4.6. Statistical Analysis

IBM SPSS Statistics software (version 24) was used as a statistical analysis tool. For the results of HPLC analysis, Levene’s test with one-way ANOVA was used to determine the significant differences among the values of metabolite contents for developmental stages in each accession. Correlations between the contents of the metabolites and the expression levels of genes related to dhurrin metabolism for the tested accessions were calculated by Pearson’s correlation. The correlation coefficients were calculated using the mean values of each point according to different developmental stages.

### 4.7. Library Construction and Sequencing

Four representative accessions (BTx623, *S. halepense*, SAP-336, and -354) with three developmental stages (seedling, vegetative, and ripening) were used for RNAseq analysis. The libraries were prepared for 101 bp paired-end sequencing using TruSeq RNA Sample Preparation Kit (Illumina, San Diego, CA, USA). Namely, mRNA molecules were purified and fragmented from 2 μg of total RNA using oligo (dT) magnetic beads. The fragmented mRNAs were synthesized as single-stranded cDNAs through random hexamer priming. By applying this as a template for second strand synthesis, double-stranded cDNA was prepared. After sequential process of end repair, A-tailing and adapter ligation, cDNA libraries were amplified with PCR. Quality of these cDNA libraries was evaluated with the Agilent 2100 BioAnalyzer (Agilent, Santa Clara, CA, USA). They were quantified with the KAPA library quantification kit (Kapa Biosystems, Wilmington, MA, USA) according to the manufacturer’s library quantification protocol. Following cluster amplification of denatured templates, sequencing was progressed as paired-end (2 × 101 bp) using Illumina HiSeq2500 (Illumina, San Diego, CA, USA). A total of 72 Gbp high quality reads from 12 samples were generated. The raw RNAseq data used and analyzed during the study are available in the NCBI SRA, https://www.ncbi.nlm.nih.gov/sra (accession no. SRX3158137−SRX3158148).

### 4.8. Transcript Assembly and Abundance Estimation Using Cufflinks

The RNAseq reads were mapped to the latest sorghum reference genome (*S. bicolor* v3.1.1) using a spliced aligner called TopHat with default parameters. The aligned read files generated by TopHat were inputted to the Cufflinks version 2.2.1 [47,48]. Reads were assembled into transcripts and their abundance were estimated. Cufflinks constructs a minimum set of transcripts that bests describe the RNAseq reads in the dataset. Cufflinks uses the normalized RNAseq fragment counts to measure the relative abundances of transcripts. The FPKM was used the unit of measurement.

### 4.9. Analysis of SNPs Associated with the Candidate Genes

The latest sorghum reference genome (*S. bicolor* v3.1.1) was downloaded from the Phytozome database (https://www.phytozome.net/) and indexed with Burrows-Wheeler Aligner (BWA) [49]. For sequence analysis and genotyping SNPs, 2-pass Spliced Transcripts Alignment to a Reference (2-pass STAR) software [50], picard software version 2.10.10 (http://broadinstitute.github.io/picard/), and the Genome Analysis Toolkit (GATK, v3.8-0) were used [51], following the GATK best practices workflow for SNP calling in RNAseq data. The 2-pass STAR detects splice junctions in first run by aligning sequence to a reference genome, and generates final alignment based on first run’s results. Picard (v2.10.10) was used to remove PCR duplicates and GATK Haplotype Caller (GATK, v3.8-0) was used to call SNPs from 12 sorghum samples and resulting in a variant call format (VCF) file. To reduce the false discovery rate, SNPs with a fisher strand (FS) less than 30 and quality by depth (QD) less than 2 were excluded.

### 4.10. Annotation

SnpEff was used to annotate and predict of calling SNP effects [23]. SnpEff is a variant annotation and effect prediction tool. It annotates and predicts the effects of genetic variants based on their genomic locations and projected coding effects. Annotated genomic locations include intronic, untranslated, upstream, downstream, splice site, or intergenic regions. Coding effects including synonymous or non-synonymous amino acid changes, start/stop codons, or frame shifts can be predicted using the SnpEff.

Predicted effects are categorized by high impact (affecting splice-sites, stop and start codons), moderate impact (non-synonymous), low impact (synonymous coding/start/stop, start gained), and modifier impact (upstream, downstream, intergenic, UTR). SNPs for 12 genes of interest were annotated from all the samples with three developmental stages. Those selected 12 genes were listed in the Table S2.

## 5. Conclusions

Sorghum is an excellent alternative crop that can grow in a harsh environment. Sorghum breeding is an important process for future use as feed. We revealed information on profiles of the metabolites; dhurrin, pHB, and pHPAAc, and gene expressions related to dhurrin metabolism for several sorghum accessions. The correlations between the metabolite contents and the gene expression levels and the comparative study would supply a clue to understanding dhurrin metabolism. The results provide future directions for the research of dhurrin metabolism as well as a background for the sorghum line breeding.

## Figures and Tables

**Table 1:** Pearson’s correlation coefficients of metabolite contents and gene expression levels.

	Dhurrin	pHB	pHPAAc	*CYP79A1*	*CYP71E1*	*UGT85B1*	*Dhr1*	*Dhr2*	*Dhr3*	*Dhr4*	*HNL1*	*NIT4A*	*NIT4B1*	*NIT4B2*	*CAS1*	*GSTL1*
pHB	0.071															
pHPAAc	0.232	0.212														
*CYP79A1*	−0.103	0.534 **	0.185													
*CYP71E1*	−0.098	0.419 **	0.107	0.956 **												
*UGT85B1*	0.019	0.206	0.043	0.588 **	0.690 **											
*Dhr1*	−0.112	0.363 **	0.284 *	0.757 **	0.685 **	0.412 **										
*Dhr2*	−0.020	0.639 **	0.253	0.639 **	0.519 **	0.191	0.650 **									
*Dhr3*	0.001	0.752 **	0.270	0.505 **	0.364 **	0.184	0.521 **	0.762 **								
*Dhr4*	−0.124	0.696 **	−0.042	0.565 **	0.496 **	0.146	0.373 **	0.497 **	0.503 **							
*HNL1*	−0.103	0.463 **	0.180	0.577 **	0.454 **	0.246	0.464 **	0.582 **	0.617 **	0.433 **						
*NIT4A*	−0.282 *	0.539 **	0.239	0.348 *	0.261	0.016	0.323 *	0.649 **	0.486 **	0.411 **	0.290 *					
*NIT4B1*	0.173	−0.069	−0.098	0.111	0.117	0.155	−0.006	0.064	−0.009	−0.062	0.273	−0.132				
*NIT4B2*	−0.092	0.470 **	0.387 **	0.414 **	0.305 *	0.215	0.504 **	0.687 **	0.546 **	0.345 *	0.466 **	0.758 **	0.048			
*CAS1*	−0.173	0.635 **	0.140	0.534 **	0.490 **	0.380 **	0.498 **	0.475 **	0.524 **	0.531 **	0.383 **	0.571 **	0.119	0.575 **		
*GSTL1*	−0.049	0.394 **	0.105	0.017	−0.015	−0.093	0.140	0.480 **	0.378 **	0.115	0.012	0.548 **	0.002	0.481 **	0.378 **	
*GSTL2*	0.145	−0.253	−0.081	−0.392 **	−0.330 *	−0.100	−0.352 *	−0.234	−0.195	−0.394 **	−0.357 *	−0.218	−0.037	−0.326 *	−0.284 *	0.195

* significant at *p* < 0.05, ** significant at *p* < 0.01.

**Table 2:** Significant single nucleotide polymorphisms (SNPs) related to dhurrin metabolism by representative sorghum accessions.

Location of SNP Marker	Reference	Substitute	Related Gene	Position	Associated Accession
Chr4:57544430	C	T	*NIT4B2*	4th intron	*S. halepense*, SAP-336, SAP-354
Chr6:2666100	C	T	*CAS1*	5′ UTR	*S. halepense*, SAP-336
Chr6:2666092	CCACCAAAG	G	*CAS1*	5′ UTR	*S. halepense*
Chr6:2666088	TGA	A	*CAS1*	5′ UTR	*S. halepense*
Chr6:2666085	CT	T	*CAS1*	5′ UTR	*S. halepense*
Chr6:2666028	GCTAGT	T	*CAS1*	5′ UTR	SAP-336
Chr6:2666028	GCTAAGCTAGT	T	*CAS1*	5′ UTR	*S. halepense*
Chr6:2665933	G	ACGG	*CAS1*	1st exon	*S. halepense*
Chr6:2665870	GC	C	*CAS1*	1st exon	*S. halepense*
Chr6:2665867	GC	C	*CAS1*	1st exon	*S. halepense*
Chr6:2665864	AGC	C	*CAS1*	1st exon	*S. halepense*
Chr6:2665840	G	A	*CAS1*	1st exon	*S. halepense*
Chr6:2665335	G	T	*CAS1*	2nd exon	*S. halepense*
Chr6:2665167	C	AGC	*CAS1*	2nd intron	SAP-336
Chr6:2665136	A	GTTAA	CAS1	2nd intron	*S. halepense*

## Supplementary Materials

The following are available online at https://www.mdpi.com/2223-7747/9/10/1390/s1, Figure S1: HPLC chromatogram showing the analysis of standard compounds, Figure S2. Dhurrin contents for each accession by different developmental stages, Figure S3. pHB contents for each accession by different developmental stages, Figure S4. pHPAAc contents for each accession by different developmental stages, Figure S5. Metabolite analysis by different developmental stages, Figure S6. Expression patterns of candidate genes for dhurrin metabolism according to different developmental stages in *S. halepense*, Figure S7. Expression patterns of candidate genes for dhurrin metabolism according to different developmental stages in Chuncheonjaerae, Figure S8. Expression patterns of candidate genes for dhurrin metabolism according to different developmental stages in Chosachal, Figure S9. Expression patterns of candidate genes for dhurrin metabolism according to different developmental stages in Banwoldang, Figure S10. Expression patterns of candidate genes for dhurrin metabolism according to different developmental stages in SAP-016, Figure S11. Expression patterns of candidate genes for dhurrin metabolism according to different developmental stages in SAP-027, Figure S12. Expression patterns of candidate genes for dhurrin metabolism according to different developmental stages in SAP-034, Figure S13. Expression patterns of candidate genes for dhurrin metabolism according to different developmental stages in SAP-115, Figure S14. Expression patterns of candidate genes for dhurrin metabolism according to different developmental stages in SAP-183, Figure S15. Expression patterns of candidate genes for dhurrin metabolism according to different developmental stages in SAP-213, Figure S16. Expression patterns of candidate genes for dhurrin metabolism according to different developmental stages in SAP-233, Figure S17. Expression patterns of candidate genes for dhurrin metabolism according to different developmental stages in SAP-265, Figure S18. Expression patterns of candidate genes for dhurrin metabolism according to different developmental stages in SAP-272, Figure S19. Expression patterns of candidate genes for dhurrin metabolism according to different developmental stages in SAP-317, Figure S20. Expression patterns of candidate genes for dhurrin metabolism according to different developmental stages in SAP-336, Figure S21. Expression patterns of candidate genes for dhurrin metabolism according to different developmental stages in SAP-354, Figure S22. Expression profiles of candidate genes related to dhurrin metabolism in BTx623 using RNAseq data, Figure S23. Expression profiles of candidate genes related to dhurrin metabolism in *S. halepense* using RNAseq data, Figure S24. Expression profiles of candidate genes related to dhurrin metabolism in SAP-336 using RNAseq data, Figure S25. Expression profiles of candidate genes related to dhurrin metabolism in SAP-354 using RNAseq data, Figure S26. Raw sequence of CAS1 by SNP analysis. Table S1. Sorghum accessions used in this study, Table S2. Genes involved in dhurrin metabolism, Table S3. Average coverage for the bam file, Table S4. Summary of analysis for SNPs related to dhurrin biosynthesis and catabolism by representative sorghum accessions, Table S5. Primers used in qRT-PCR.
