# Supplementary material for: Prediction of Dhurrin Metabolism by Transcriptome and Metabolome Analyses in Sorghum
Source: Plants (Basel). 2020 Oct 19;9(10):1390. doi: 10.3390/plants9101390 (PMC7589853; doi:10.3390/plants9101390)
Supplement: Supplementary file 1 [file plants-09-01390-s001.pdf]

Table S1. Sorghum accessions used in this study.

Accession	Information	Additional classification
BTx623	IT 148333	reference
S. halepense	Polyploid	wild species
Chuncheonjaerae	IT 028133	local variety
Chosachal	IT 028134	local variety
Banwoldang	IT 028135	local variety
SAP-016	Ajabsido	
SAP-027	Tx378	
SAP-034	Tx631	
SAP-115	Tx430	milo/feterita
SAP-183	SC1451	
SAP-213	SC283	guinea/caudatum
SAP-233	SC35	durra
SAP-265	SC55	
SAP-272	SC566-14 (IS 7254C)	
SAP-317	SC704	
SAP-336	SC855	
SAP-354	Segaolane	kafir

Table S2. Genes involved in dhurrin metabolism.

Pathway	Gene	Locus number	Location	Description	Reference
**Biosynthesis**	*CYP79A1*	Sb01g001200	Chr01:1144011..1146367 forward	similar to Cytochrome P450 79A1	Bak *et al.* 1998
	CYP71E1	Sb01g001180	Chr01:1048825..1050688 reverse	similar to Cytochrome P450 71E1	Bak *et al.* 1998
	UGT85B1	Sb01g001220	Chr01:1152501..1153979 forward	similar to UDP-glucose glucosyltransferase	Jones *et al.* 1999
**Catabolism**	*Dhr1*	Sb08g007570	Chr08:13362295..13366903 forward	similar to Dhurrinase	Hösel *et al.* 1987; Cicek and Esen 1998
	Dhr2	Sb08g007610	Chr08:13754078..13757147 reverse	similar to Cyanogenic beta-glucosidase2 dhurrinase-2	Hösel *et al.* 1987; Cicek and Esen 1998
	Dhr3	Sb08g007586	Chr08:13555583..13585887 forward	similar to Dhurrinase	Mizuno *et al.* 2012
	Dhr4	Sb08g007650	Chr08:13985353..13991301 forward	similar to Dhurrinase	Mizuno *et al.* 2012
	HNL1	Sb04g036350	Chr04:66773685..66780194 forward	similar to P-(S)-hydroxymandelonitrile lyase precursor (EC 4.1.2.11) (Hydroxynitrile lyase) (HNL)	Wajant *et al.* 1994
	NIT4A	Sb04g026950	Chr04:57551584..57555520 forward	similar to Nitrilase 2	Jenrich *et al.* 2007
	NIT4B1	Sb04g026930	Chr04:57535766..57539748 forward	similar to Nitrilase 1	Jenrich *et al.* 2007
	NIT4B2	Sb04g026940	Chr04:57540808..57544914 forward	similar to Putative nitrilase 1	Jenrich *et al.* 2007
	GSTL1	Sb02g043250	Chr02:76854067..76856024 reverse	similar to IN2-1 protein	Bjarnholt *et al.* 2018
	GSTL2	Sb09g002800	Chr09:3000494..3003302 reverse	similar to IN2-1 protein	Bjarnholt *et al.* 2018
**Detoxification**	*CAS1*	Sb06g001610	Chr06:2661605..2666186 reverse	similar to Cysteine synthase	Wurtele *et al.* 1984

Table S3. Average coverage for the bam file.

Sample (accession/developmental stage)	Average coverage
BTx623/seedling stage	40.2512
BTx623/vegetative stage	41.2614
BTx623/ripening stage	27.8931
S.halepense/seedling stage	42.631
S.halepense/vegetative stage	38.5091
S.halepense/ripening stage	32.3872
SAP-336/seedling stage	49.5892
SAP-336/vegetative stage	28.7789
SAP-336/ripening stage	25.2882
SAP-354/seedling stage	47.2692
SAP-354/vegetative stage	40.6327
SAP-354/ripening stage	19.0372

Table S4. Summary of analysis for SNPs related to dhurrin biosynthesis and catabolism by representative sorghum accessions.

Sample (accession/ developmental stage)	Total SNP number	Significant SNP number[1]	SNPs within each accession[2]	Total SNPs across accessions[3]
BTx623/seedling stage	12	0		
BTx623/vegetative stage	1	0	0	
BTx623/ripening stage	0	0		
S.halepnese/seedling stage	781	12		
S.halepnese/vegetative stage	675	11	13	
S.halepnese/ripening stage	506	14		15
SAP-336/seedling stage	225	5		
SAP-336/vegetative stage	115	3	4	
SAP-336/ripening stage	68	2		
SAP-354/seedling stage	182	2		
SAP-354/vegetative stage	71	0	1	
SAP-354/ripening stage	65	0		

[1]High effect SNPs predicted by SnpEff.

[2]Remove overlapped SNPs in each accession independently of developmental stage.

[3]Remove overlapped SNPs across representative accessions.

Table S5. Primers used in qRT-PCR.

Primer	Sequence (5' to 3')
PP2A_F	CAAATTGAGCCAGACACAAC
PP2A_R	AAAGCAGCAACAAAATCTCC
CYP79A1_F	TGCTCATCACTCTCAAGGAC
CYP79A1_R	TGTCCGACTCCTGCACTAG
CYP71E1_F	TCATCAACCTGTGCAAGGAG
CYP71E1_R	TGACCACCATCTTCAGGTAC
UGT85B1_F	CGACGATTACCTGGACACG
UGT85B1_R	GCCAGATGCTGATGTCCATG
Dhr1_F	GATAAGCAGTGAGTCTGCTG
Dhr1_R	ACCTGACATCCTCAGCGTAC
Dhr2_F	GACGACCACATAAGGCTAG
Dhr2_R	TACTACGGGTAAACATCAGC
Dhr3_F	AGCAGAACTTAGAAGCCATC
Dhr3_R	TCAGCATGTGGATTCCAGG
Dhr4_F	CCATATGTACGCTGACGATG
Dhr4_R	CTGTGTAATCTGTTATAATCCTG
HNL1_F	GCAGTCTTCAACTCCATCAAC
HNL1_R	CCTGTCCCCACTGATCAAAT
NIT4A_F	ACGATTAGCTGCTATGGCTG
NIT4A_R	ACAGGAATAGTCGATCCATC
NIT4B1_F	CCTTGGAGAGATTGTTCGAG
NIT4B1_R	GTCTGCGACTACTTTCATGC
NIT4B2_F	GTCTCTTTCACTGCTTCAGC
NIT4B2_R	GATCGATCTCATATCTGTAGG
CAS1_F	CGTCAAAGACAGACCAGCAA
CAS1_R	TTCGTTGGGTCGGTAAGAAC
GSTL1_F	CAAGCAAGCTTTTGCTGATG
GSTL1_R	GAAATCCTTGAAGCGTTCGAC
GSTL2_F	GCTCTTGACAAGGTGGAGGA
GSTL2_R	AGGTCGCTGTGTAGGCATCT

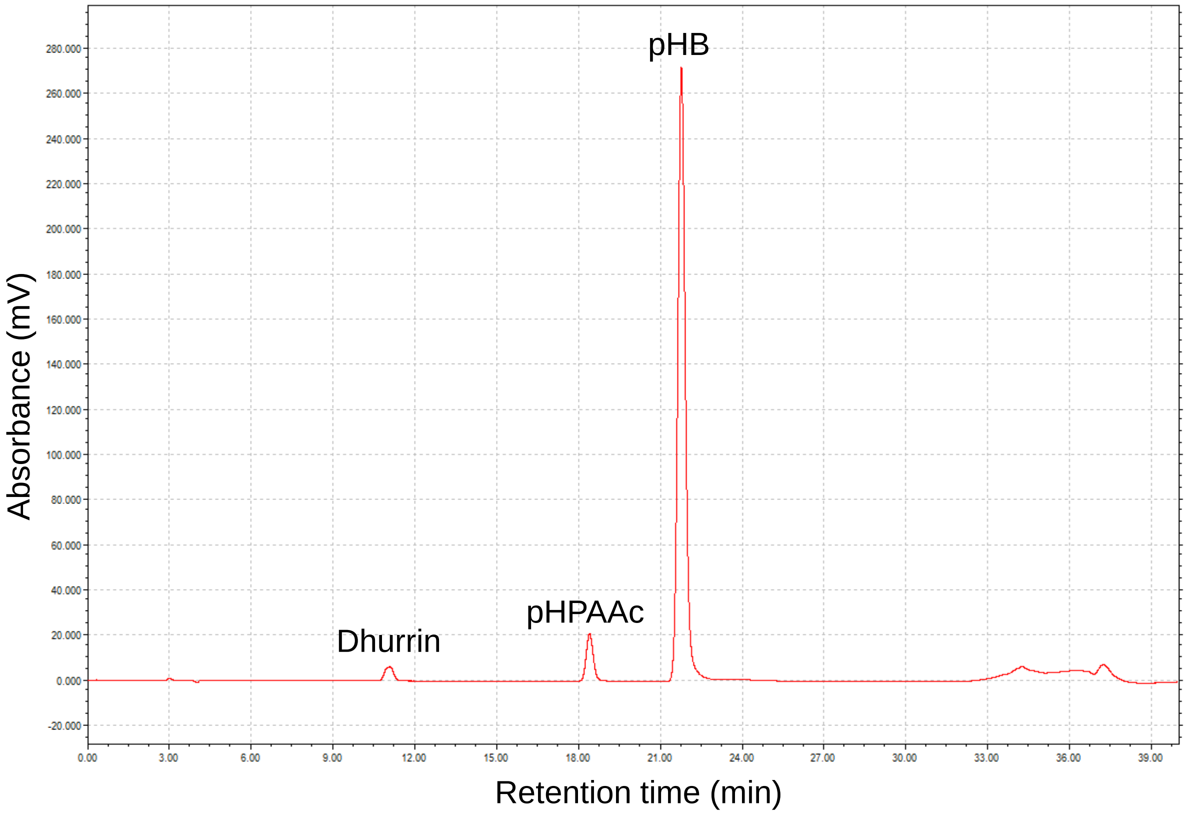

Figure S1. HPLC chromatogram showing the analysis of standard compounds. This clearly shows the good separation among dhurrin, pHB, and pHPAAc peaks. *Y*-axis, absorbance at 280 nm. *X*-axis, retention time (min). pHPAAc, *p*-hydroxyphenylacetic acid; pHB, *p*-hydroxybenzaldehyde.

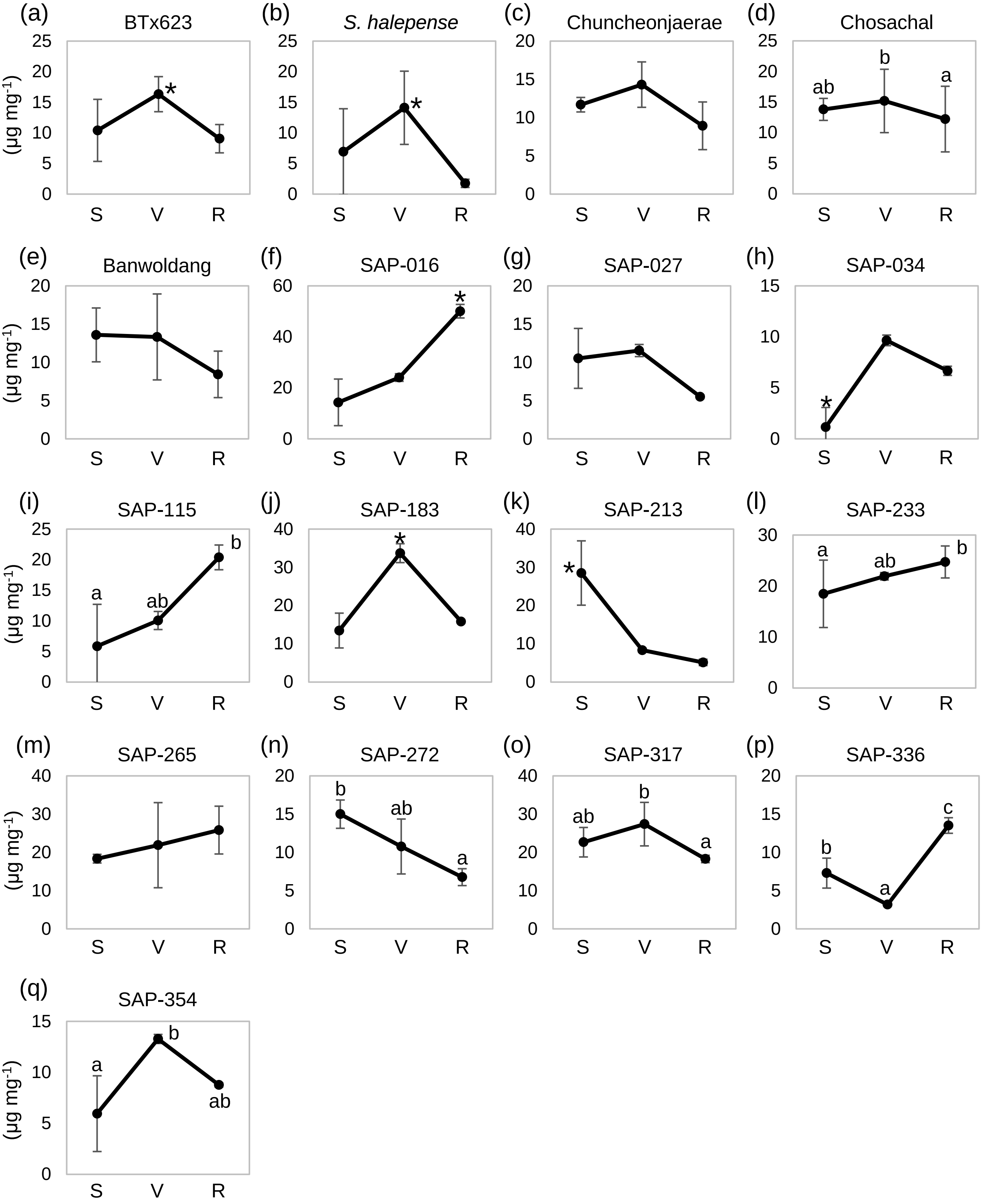

Figure S2. Dhurrin contents for each accession by different developmental stages. (a) BTx623. (b) *S. halepense*. (c) Chosachal. (d) Chuncheonjaerae. (e) Banwoldang. (f) SAP-016. (g) SAP-027. (h) SAP-034. (i) SAP-115. (j) SAP-183. (k) SAP-213. (l) SAP-233. (m) SAP-265. (n) SAP-272. (o) SAP-317. (p) SAP-336. (q) SAP-354. *Y*-axis, amount of dhurrin (μg mg^{-1}). *X*-axis, developmental stage. S, seedling stage; V, vegetative stage; R, ripening stage. Asterisks represent significant difference compared to other developmental stage(s) and lower-case letters indicate homogeneous subsets in each accession (P = 0.05). Values are shown as means. Error bars indicate standard deviation. *n* = 3 or more.

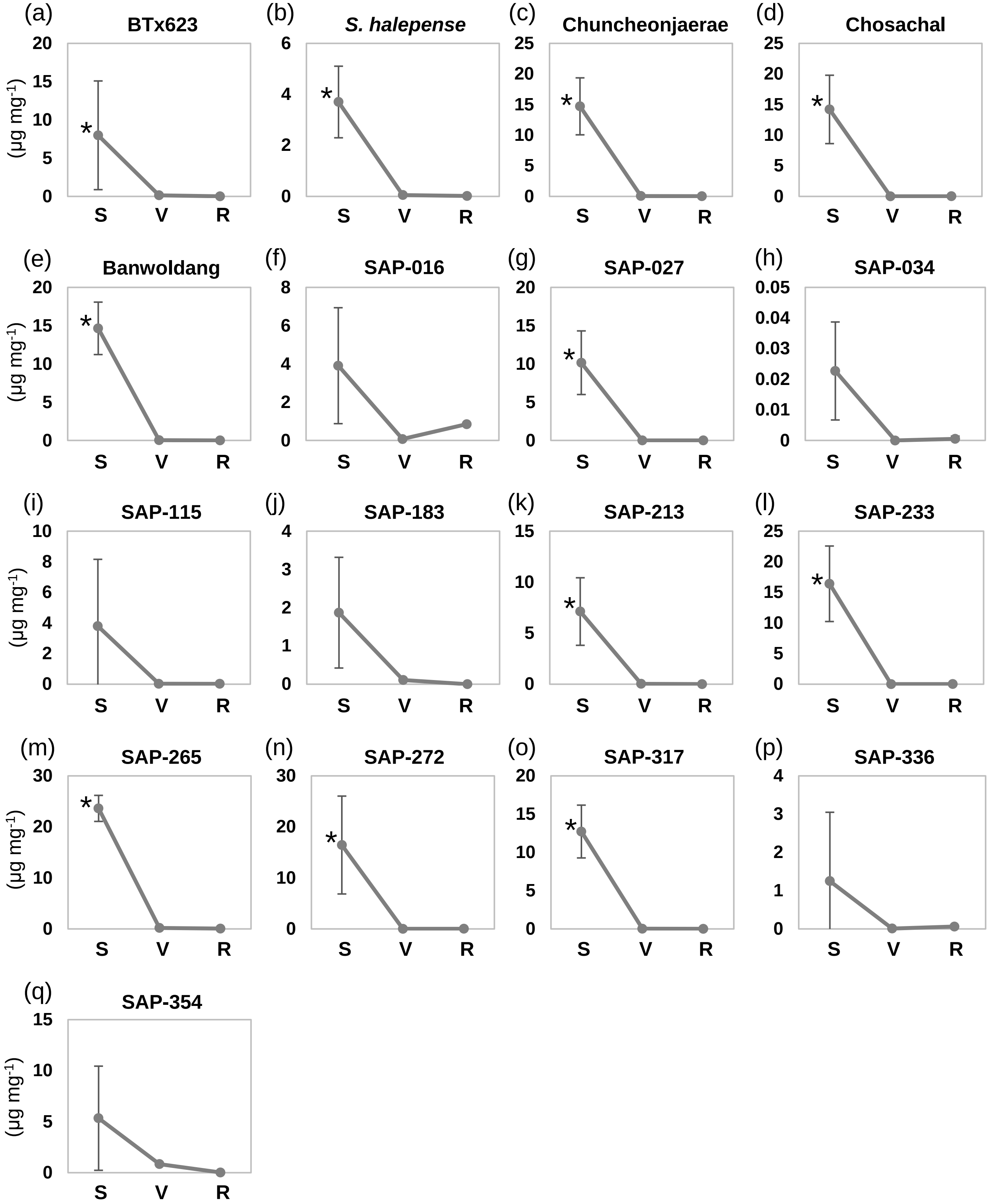

Figure S3. pHB contents for each accession by different developmental stages. (a) BTx623. (b) *S. halepense*. (c) Chosachal. (d) Chuncheonjaerae. (e) Banwoldang. (f) SAP-016. (g) SAP-027. (h) SAP-034. (i) SAP-115. (j) SAP-183. (k) SAP-213. (l) SAP-233. (m) SAP-265. (n) SAP-272. (o) SAP-317. (p) SAP-336. (q) SAP-354. *Y*-axis, amount of pHB (μg mg^{-1}). *X*-axis, developmental stage. S, seedling stage; V, vegetative stage; R, ripening stage. Asterisks represent significant difference compared to other developmental stage(s) in each accession (P = 0.05). Values are shown as means. Error bars indicate standard deviation. n = 3 or more.

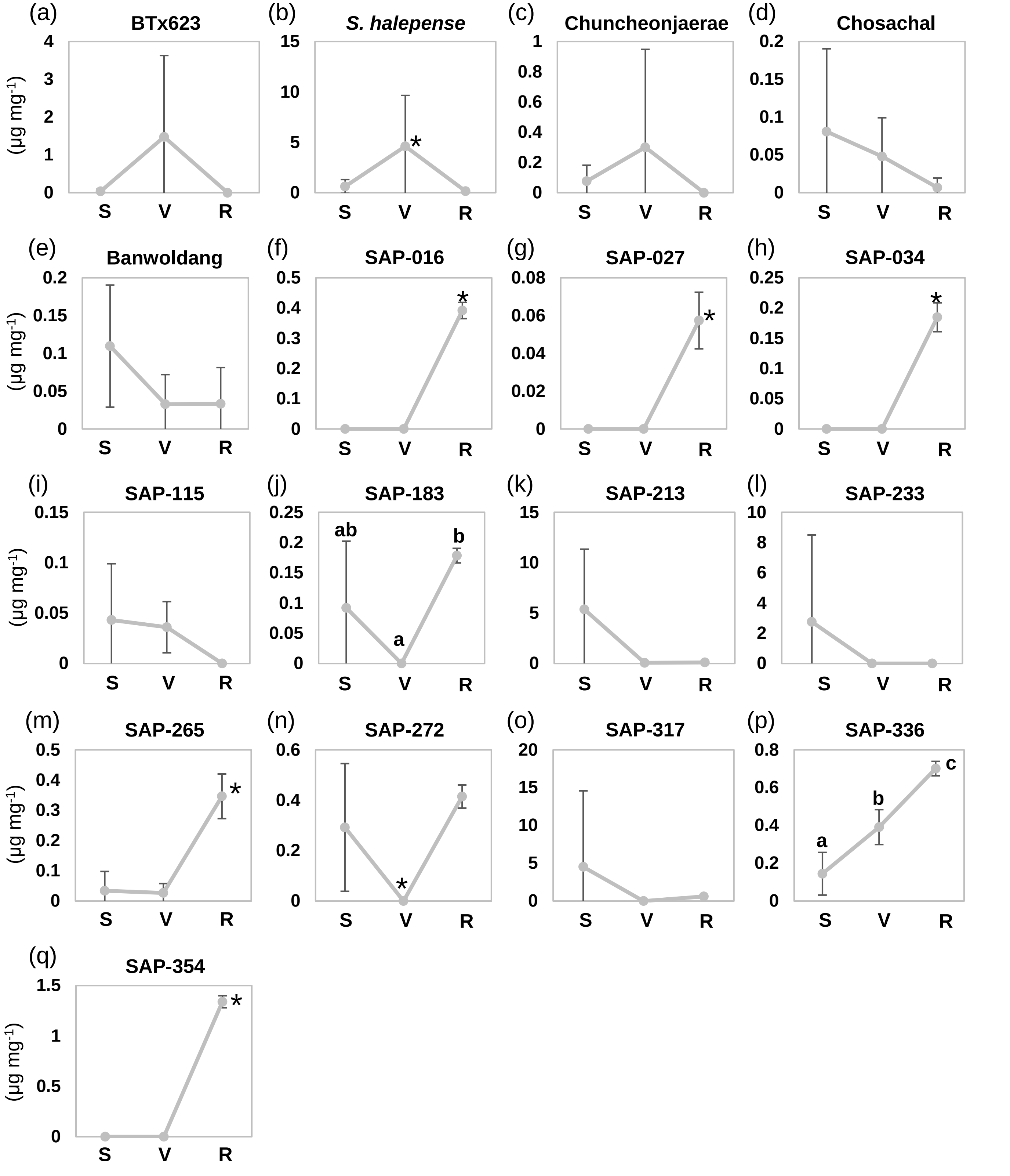

Figure S4. pHPAAc contents for each accession by different developmental stages. (a) BTx623. (b) *S. halepense*. (c) Chosachal. (d) Chuncheonjaerae. (e) Banwoldang. (f) SAP-016. (g) SAP-027. (h) SAP-034. (i) SAP-115. (j) SAP-183. (k) SAP-213. (l) SAP-233. (m) SAP-265. (n) SAP-272. (o) SAP-317. (p) SAP-336. (q) SAP-354. *Y*-axis, amount of pHPAAc (μg mg^{-1}). *X*-axis, developmental stage. S, seedling stage; V, vegetative stage; R, ripening stage. Asterisks represent significant difference compared to other developmental stage(s) and lower-case letters indicate homogeneous subsets in each accession (P = 0.05). Values are shown as means. Error bars indicate standard deviation. *n* = 3 or more.

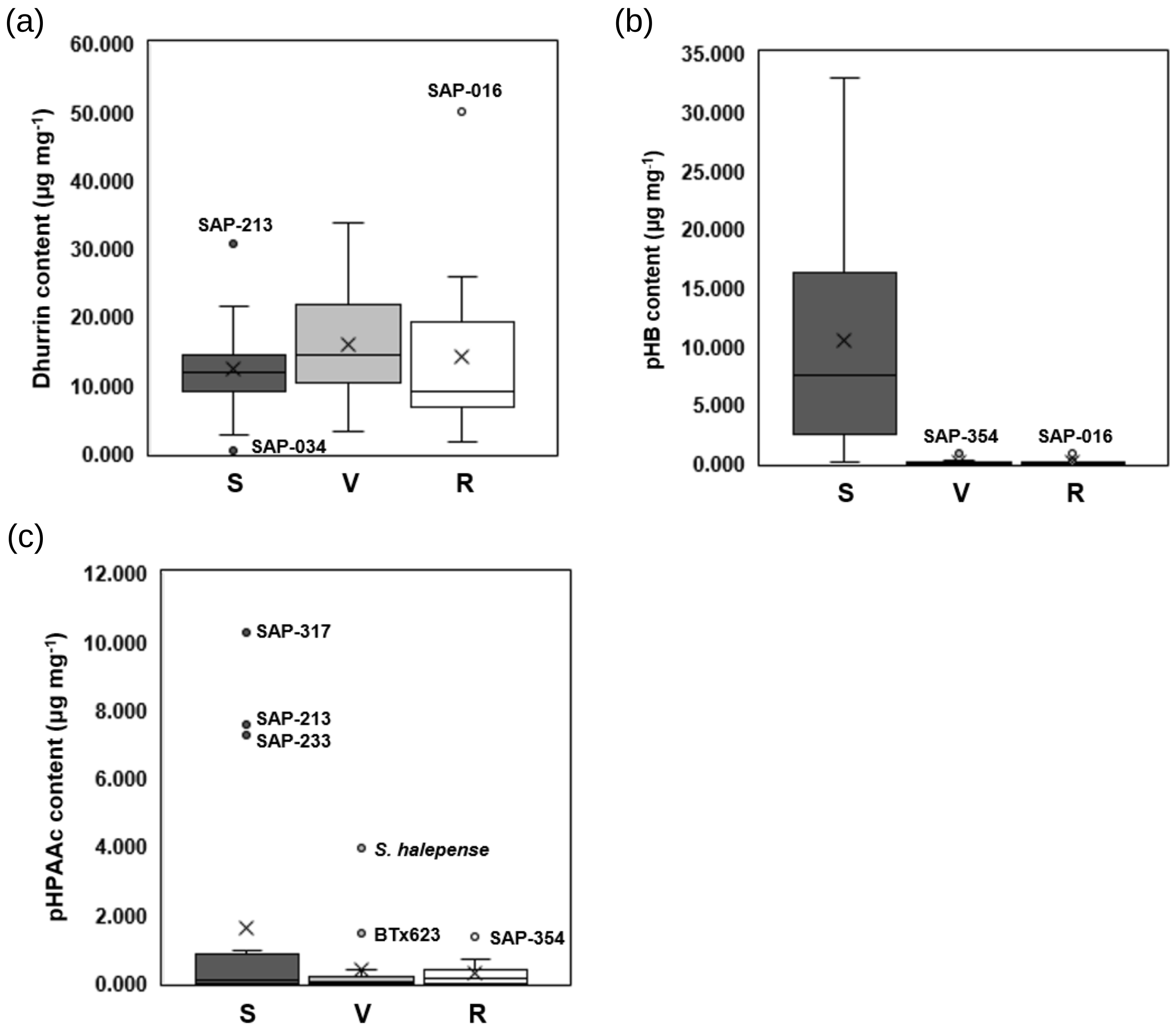

Figure S5. Metabolite analysis by different developmental stages. Box plots show mean values for contents of three metabolites in the all accessions. (a) Dhurrin. (b) *p*-Hydroxybenzaldehyde (pHB). (c) *p*-Hydroxyphenylacetic acid (pHPAAc). The Xs in box plots represents means while the band is the median in the box, and the box edges indicate the 25th and 75th percentiles. The whiskers represent the largest and smallest values that within 1.5 times the interquartile ranges, and the dots indicate outliers of the whisker range. *Y*-axis, amount of each metabolite (μg mg^{-1}). *X*-axis, developmental stages. S, seedling stage; V, vegetative stage; R, ripening stage; SAP, Sorghum association panel.

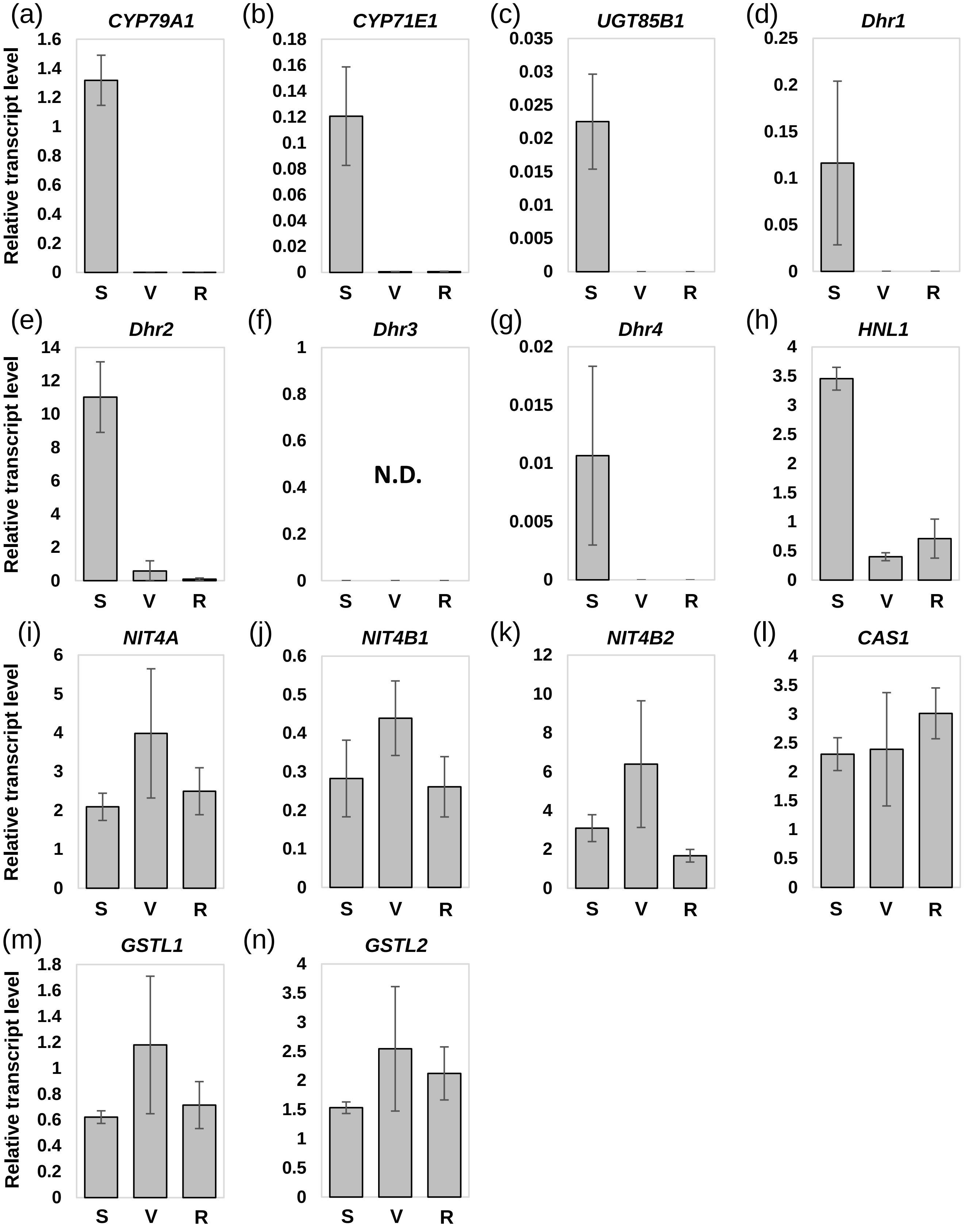

Figure S6. Expression patterns of candidate genes for dhurrin metabolism according to different developmental stages in *S. halepense*. qRT-PCR analyses of (a) *CYP79A1*, (b) *CYP71E1*, (c) *UGT85B1*, (d) *Dhr1*, (e) *Dhr2*, (f) *Dhr3*, (g) *Dhr4*, (h) *HNL1*, (i) *NIT4A*, (j) *NIT4B1*, (k) *NIT4B2*, (l) *CAS1*, (m) *GSTL1*, (n) *GSTL2*. Transcripts of *Dhr3* were not detectable in *S. halepense*. *Y*-axis, transcript level relative to sorghum *PP2A* expression. *X*-axis, developmental stage. S, seedling stage; V, vegetative stage; R, ripening stage; N.D., non-detectable. Values are shown as means. Error bars indicate standard deviation. n = 3 or more.

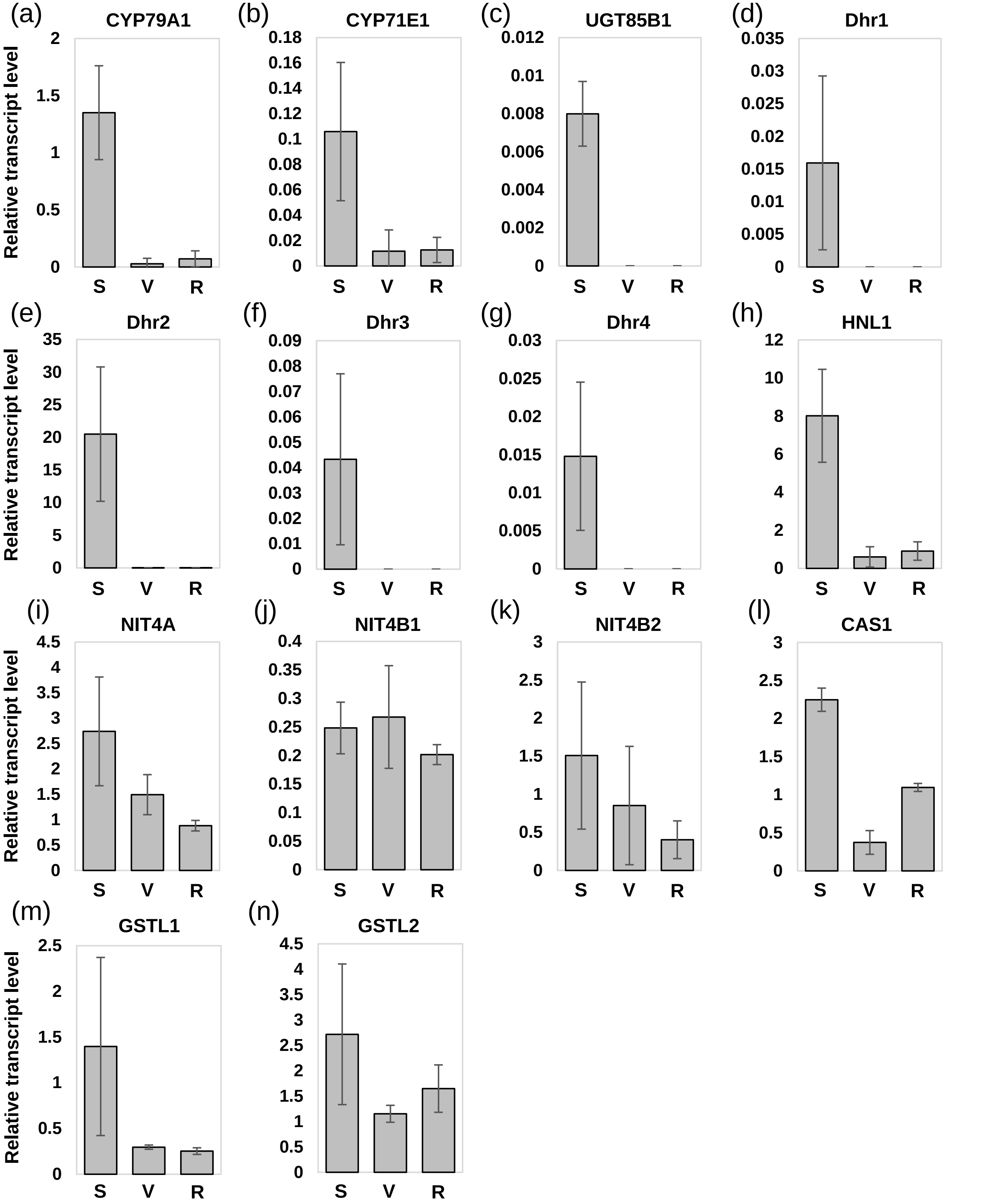

Figure S7. Expression patterns of candidate genes for dhurrin metabolism according to different developmental stages in Chuncheonjaerae. qRT-PCR analyses of (a) *CYP79A1*, (b) *CYP71E1*, (c) *UGT85B1*, (d) *Dhr1*, (e) *Dhr2*, (f) *Dhr3*, (g) *Dhr4*, (h) *HNL1*, (i) *NIT4A*, (j) *NIT4B1*, (k) *NIT4B2*, (l) *CAS1*, (m) *GSTL1*, (n) *GSTL2*. *Y*-axis, transcript level relative to sorghum *PP2A* expression. *X*-axis, developmental stages. S, seedling stage; V, vegetative stage; R, ripening stage. Values are shown as means. Error bars indicate standard deviation. $n = 3$ or more.

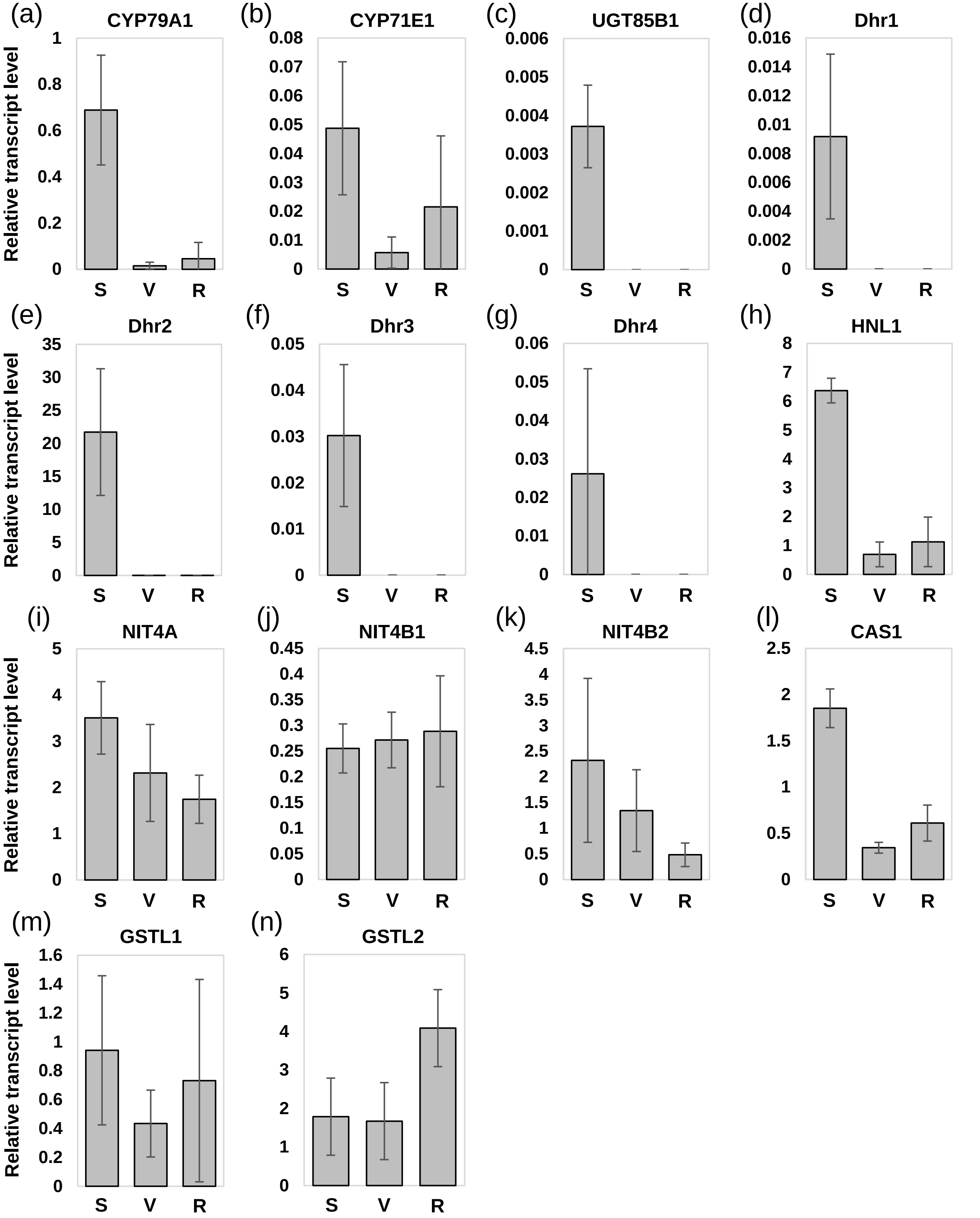

Figure S8. Expression patterns of candidate genes for dhurrin metabolism according to different developmental stages in Chosachal. qRT-PCR analyses of (a) *CYP79A1*, (b) *CYP71E1*, (c) *UGT85B1*, (d) *Dhr1*, (e) *Dhr2*, (f) *Dhr3*, (g) *Dhr4*, (h) *HNL1*, (i) *NIT4A*, (j) *NIT4B1*, (k) *NIT4B2*, (l) *CAS1*, (m) *GSTL1*, (n) *GSTL2*. *Y*-axis, transcript level relative to sorghum *PP2A* expression. *X*-axis, developmental stages. S, seedling stage; V, vegetative stage; R, ripening stage. Values are shown as means. Error bars indicate standard deviation. n = 3 or more.

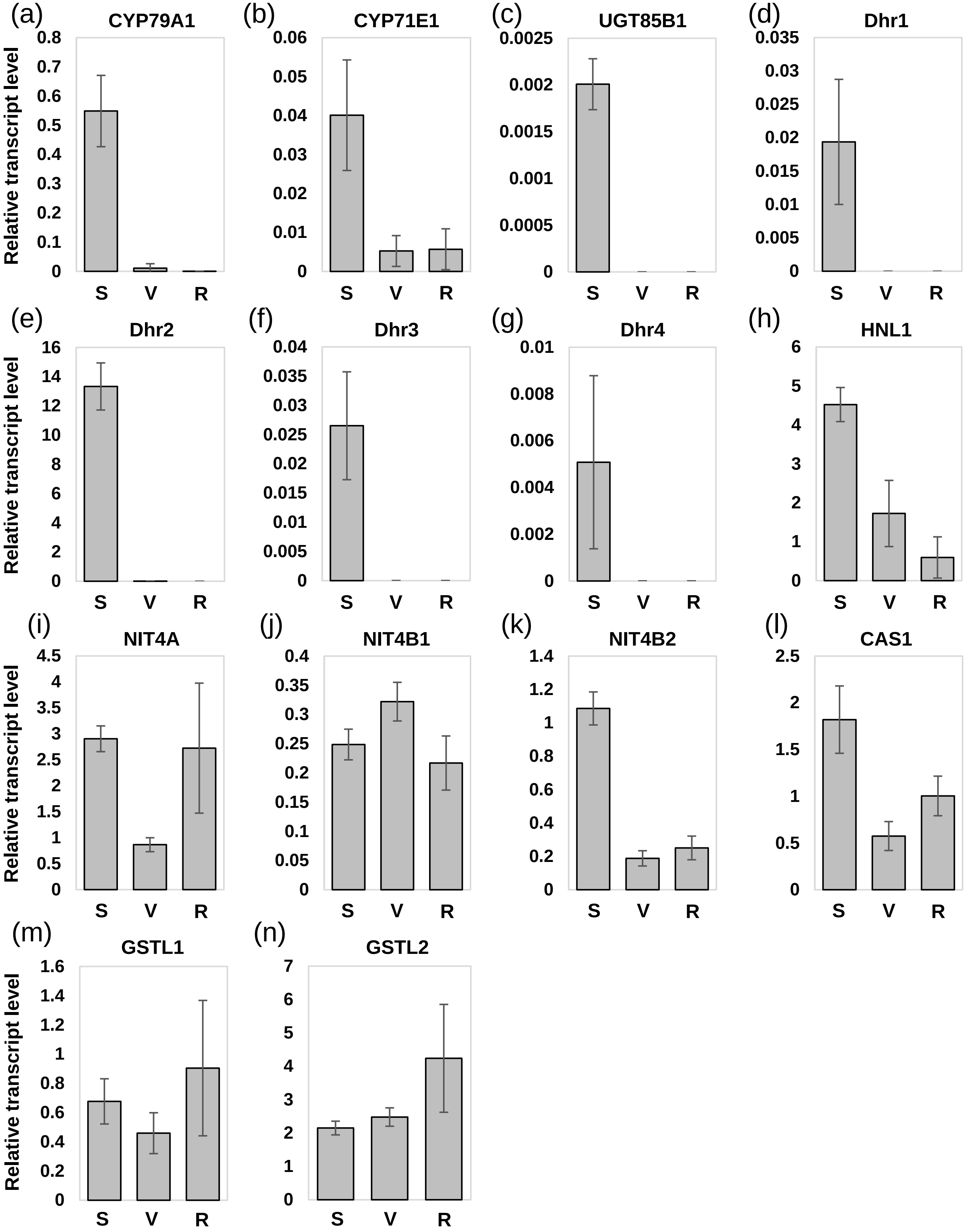

Figure S9. Expression patterns of candidate genes for dhurrin metabolism according to different developmental stages in Banwoldang. qRT-PCR analyses of (a) *CYP79A1*, (b) *CYP71E1*, (c) *UGT85B1*, (d) *Dhr1*, (e) *Dhr2*, (f) *Dhr3*, (g) *Dhr4*, (h) *HNL1*, (i) *NIT4A*, (j) *NIT4B1*, (k) *NIT4B2*, (l) *CAS1*, (m) *GSTL1*, (n) *GSTL2*. *Y*-axis, transcript level relative to sorghum *PP2A* expression. *X*-axis, developmental stages. S, seedling stage; V, vegetative stage; R, ripening stage. Values are shown as means. Error bars indicate standard deviation. *n* = 3 or more.

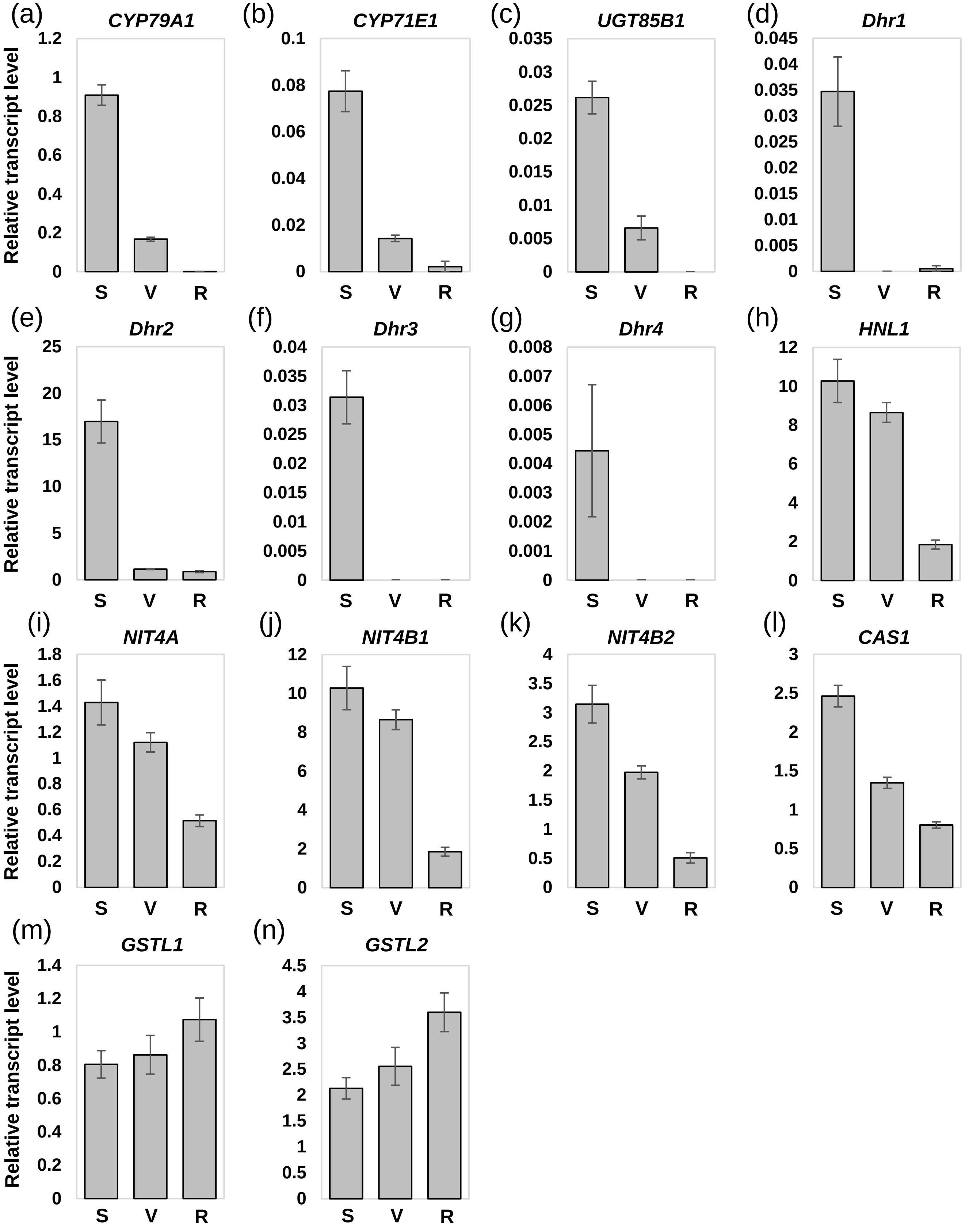

Figure S10. Expression patterns of candidate genes for dhurrin metabolism according to different developmental stages in SAP-016. qRT-PCR analyses of (a) *CYP79A1*, (b) *CYP71E1*, (c) *UGT85B1*, (d) *Dhr1*, (e) *Dhr2*, (f) *Dhr3*, (g) *Dhr4*, (h) *HNL1*, (i) *NIT4A*, (j) *NIT4B1*, (k) *NIT4B2*, (l) *CAS1*, (m) *GSTL1*, (n) *GSTL2*. *Y*-axis, transcript level relative to sorghum *PP2A* expression. *X*-axis, developmental stages. S, seedling stage; V, vegetative stage; R, ripening stage. Values are shown as means. Error bars indicate standard deviation. *n* = 3 or more.

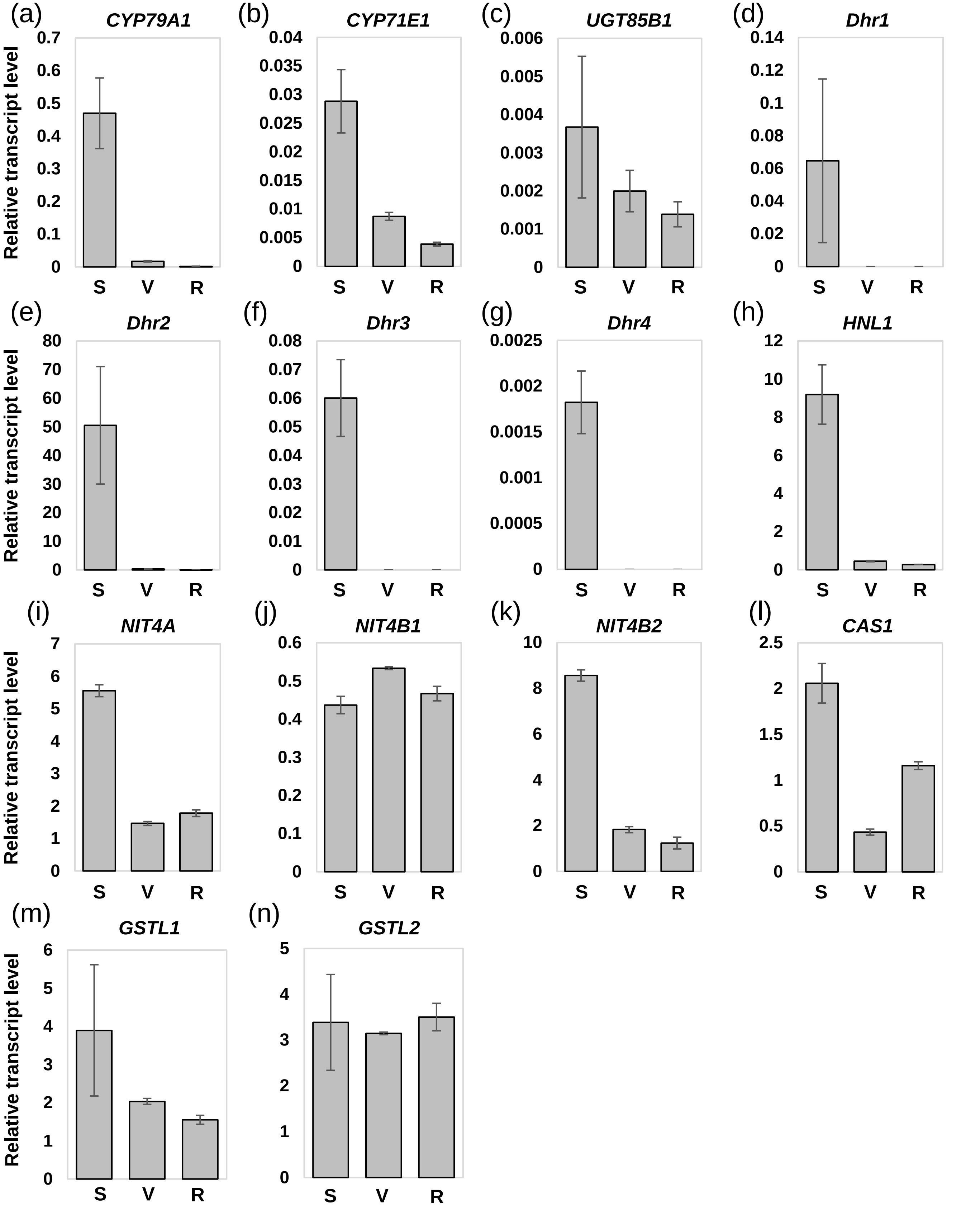

Figure S11. Expression patterns of candidate genes for dhurrin metabolism according to different developmental stages in SAP-027. qRT-PCR analyses of (a) *CYP79A1*, (b) *CYP71E1*, (c) *UGT85B1*, (d) *Dhr1*, (e) *Dhr2*, (f) *Dhr3*, (g) *Dhr4*, (h) *HNL1*, (i) *NIT4A*, (j) *NIT4B1*, (k) *NIT4B2*, (l) *CAS1*, (m) *GSTL1*, (n) *GSTL2*. *Y*-axis, transcript level relative to sorghum *PP2A* expression. *X*-axis, developmental stages. S, seedling stage; V, vegetative stage; R, ripening stage. Values are shown as means. Error bars indicate standard deviation. $n = 3$ or more.

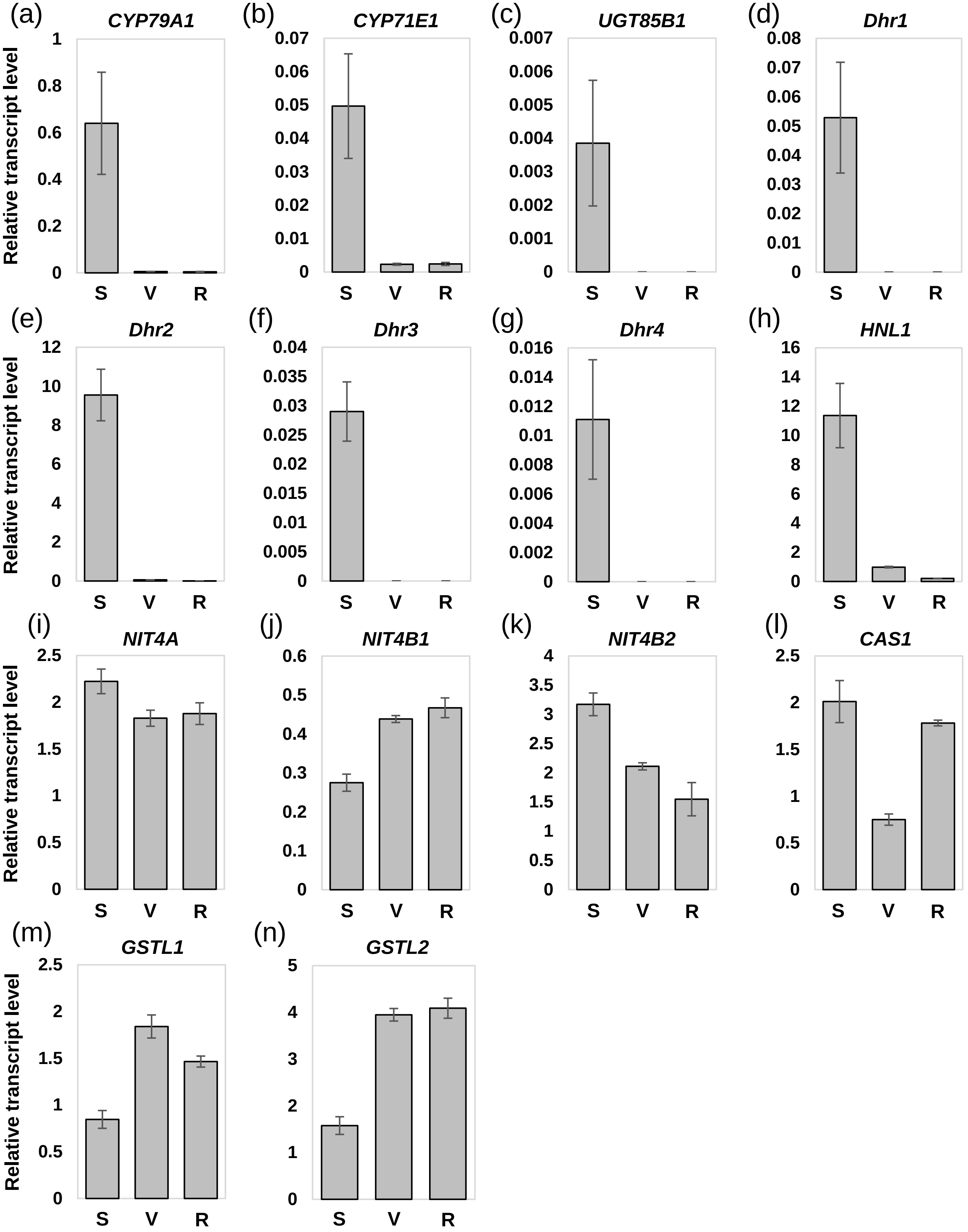

Figure S12. Expression patterns of candidate genes for dhurrin metabolism according to different developmental stages in SAP-034. qRT-PCR analyses of (a) *CYP79A1*, (b) *CYP71E1*, (c) *UGT85B1*, (d) *Dhr1*, (e) *Dhr2*, (f) *Dhr3*, (g) *Dhr4*, (h) *HNL1*, (i) *NIT4A*, (j) *NIT4B1*, (k) *NIT4B2*, (l) *CAS1*, (m) *GSTL1*, (n) *GSTL2*. *Y*-axis, transcript level relative to sorghum *PP2A* expression. *X*-axis, developmental stages. S, seedling stage; V, vegetative stage; R, ripening stage. Values are shown as means. Error bars indicate standard deviation. $n = 3$ or more.

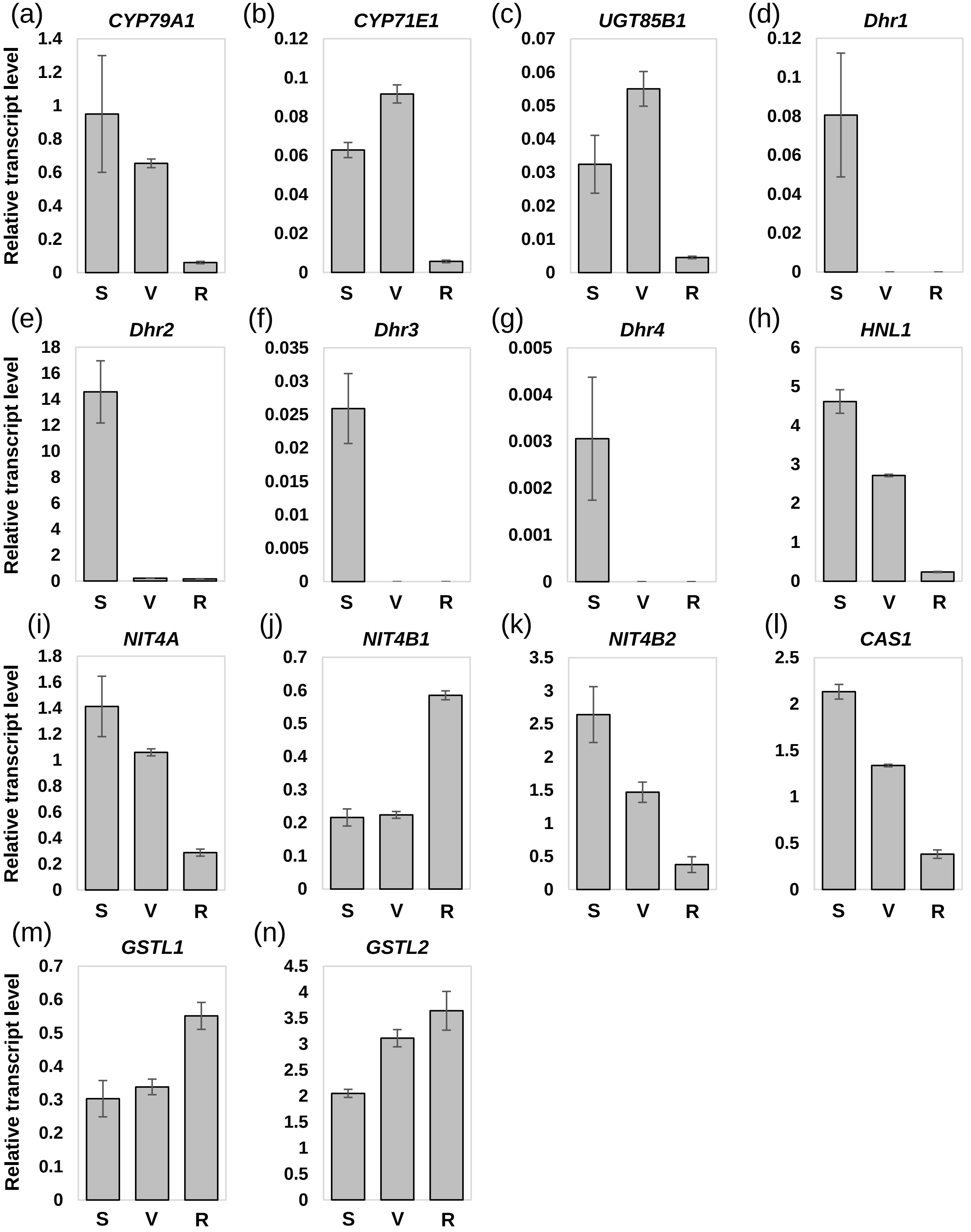

Figure S13. Expression patterns of candidate genes for dhurrin metabolism according to different developmental stages in SAP-115. qRT-PCR analyses of (a) *CYP79A1*, (b) *CYP71E1*, (c) *UGT85B1*, (d) *Dhr1*, (e) *Dhr2*, (f) *Dhr3*, (g) *Dhr4*, (h) *HNL1*, (i) *NIT4A*, (j) *NIT4B1*, (k) *NIT4B2*, (l) *CAS1*, (m) *GSTL1*, (n) *GSTL2*. *Y*-axis, transcript level relative to sorghum *PP2A* expression. *X*-axis, developmental stages. S, seedling stage; V, vegetative stage; R, ripening stage. Values are shown as means. Error bars indicate standard deviation. n = 3 or more.

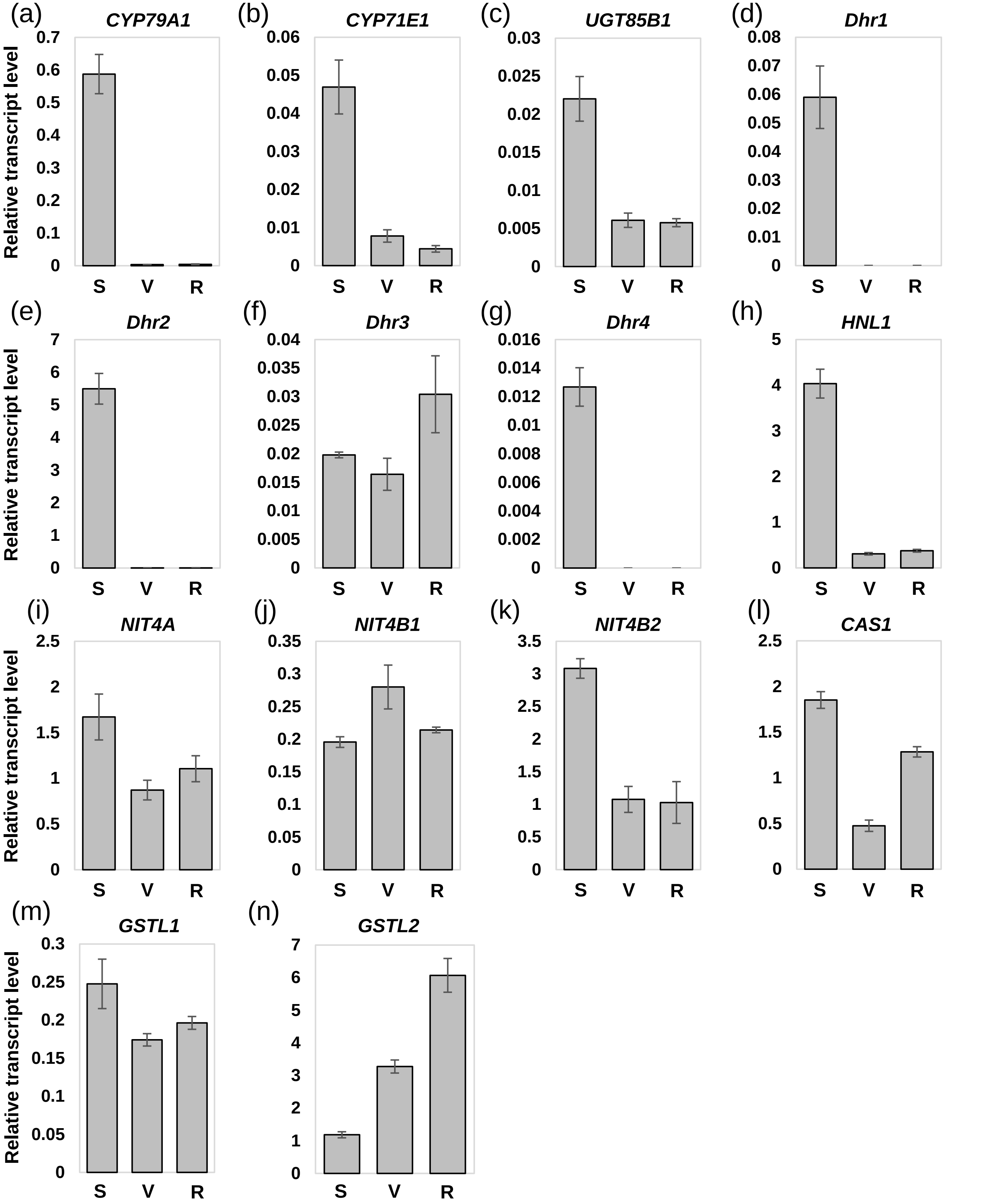

Figure S14. Expression patterns of candidate genes for dhurrin metabolism according to different developmental stages in SAP-183. qRT-PCR analyses of (a) *CYP79A1*, (b) *CYP71E1*, (c) *UGT85B1*, (d) *Dhr1*, (e) *Dhr2*, (f) *Dhr3*, (g) *Dhr4*, (h) *HNL1*, (i) *NIT4A*, (j) *NIT4B1*, (k) *NIT4B2*, (l) *CAS1*, (m) *GSTL1*, (n) *GSTL2*. *Y*-axis, transcript level relative to sorghum *PP2A* expression. *X*-axis, developmental stages. S, seedling stage; V, vegetative stage; R, ripening stage. Values are shown as means. Error bars indicate standard deviation. *n* = 3 or more.

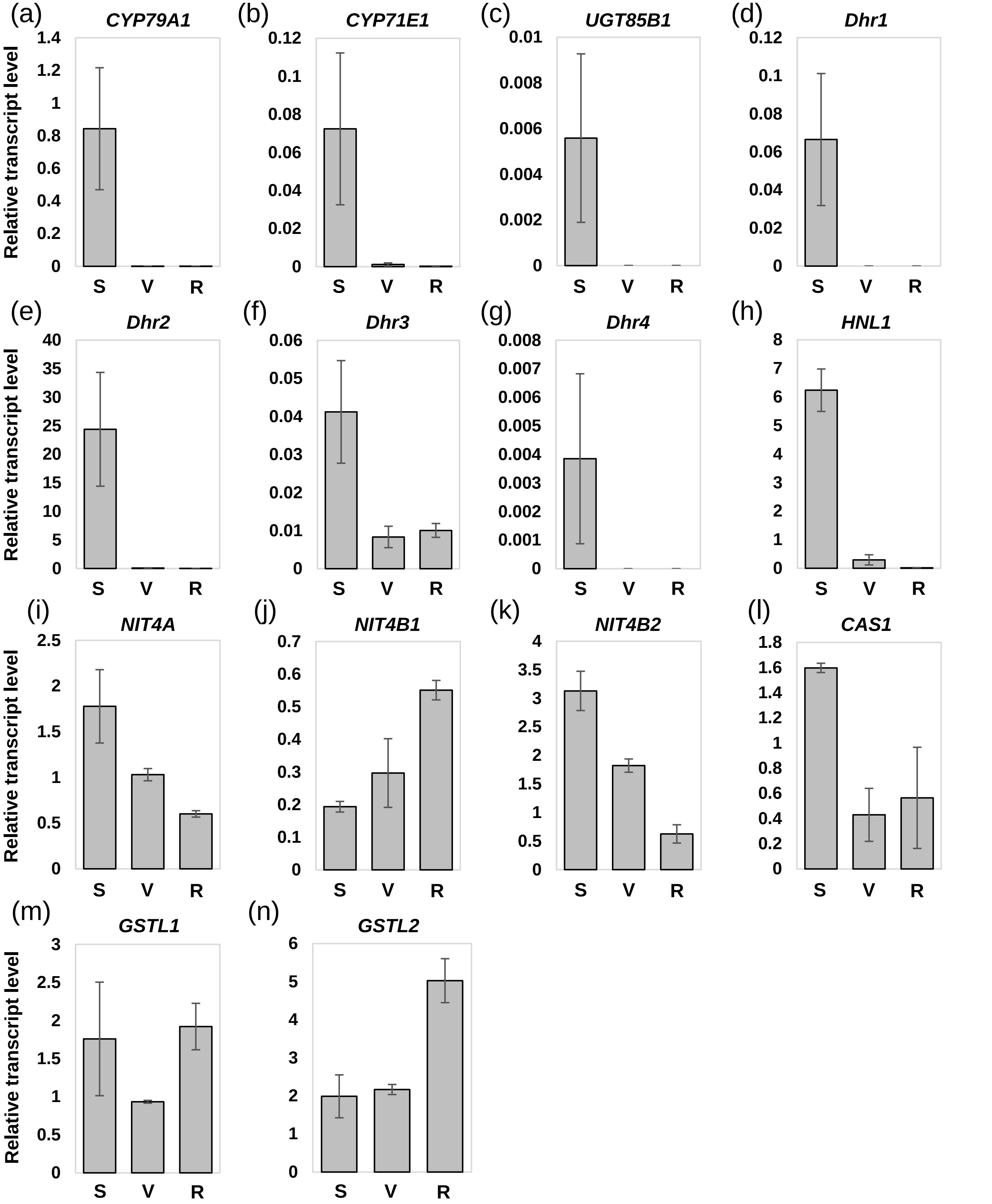

Figure S15. Expression patterns of candidate genes for dhurrin metabolism according to different developmental stages in SAP-213. qRT-PCR analyses of (a) *CYP79A1*, (b) *CYP71E1*, (c) *UGT85B1*, (d) *Dhr1*, (e) *Dhr2*, (f) *Dhr3*, (g) *Dhr4*, (h) *HNL1*, (i) *NIT4A*, (j) *NIT4B1*, (k) *NIT4B2*, (l) *CAS1*, (m) *GSTL1*, (n) *GSTL2*. *Y*-axis, transcript level relative to sorghum *PP2A* expression. *X*-axis, developmental stages. S, seedling stage; V, vegetative stage; R, ripening stage. Values are shown as means. Error bars indicate standard deviation. *n* = 3 or more.

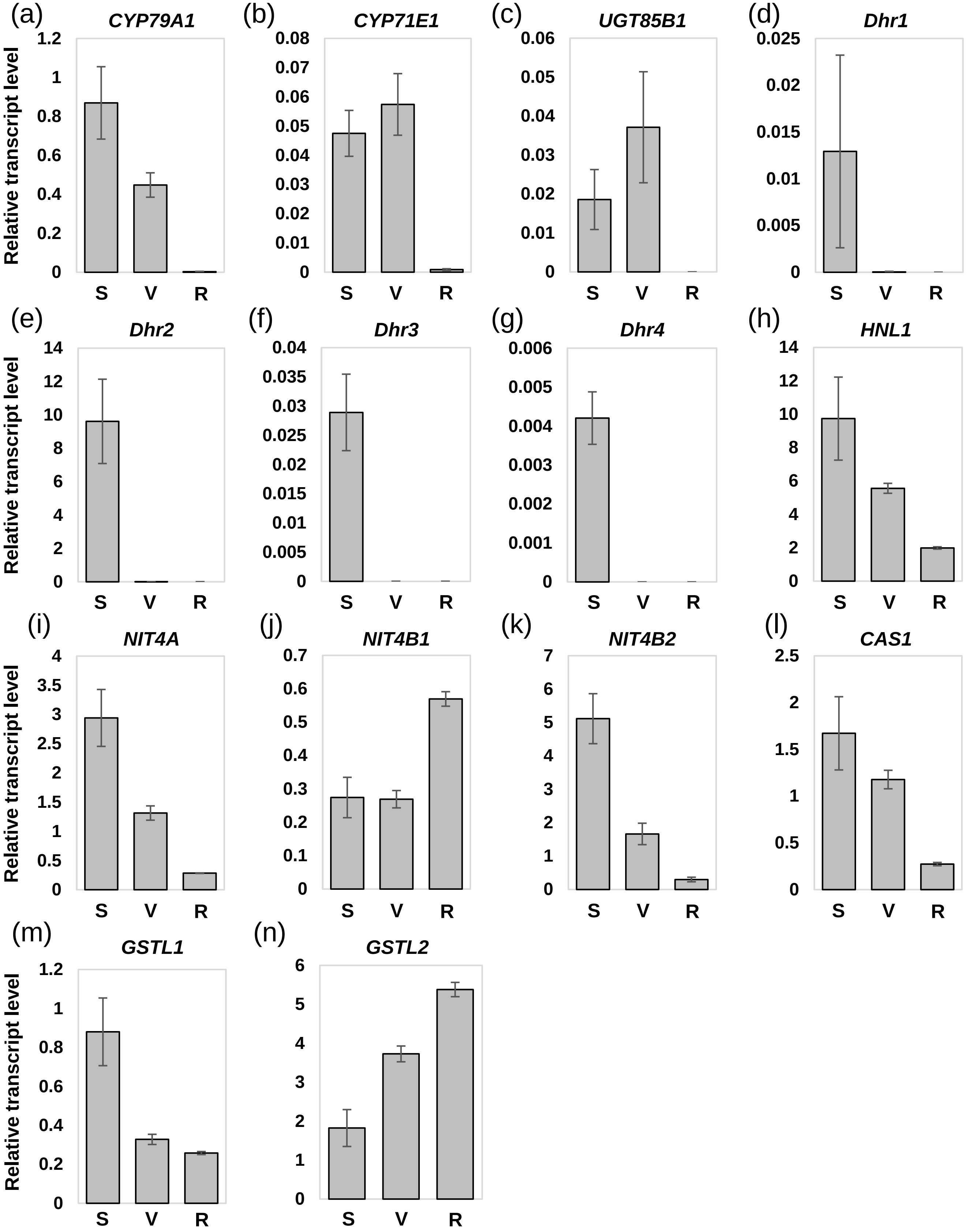

Figure S16. Expression patterns of candidate genes for dhurrin metabolism according to different developmental stages in SAP-233. qRT-PCR analyses of (a) *CYP79A1*, (b) *CYP71E1*, (c) *UGT85B1*, (d) *Dhr1*, (e) *Dhr2*, (f) *Dhr3*, (g) *Dhr4*, (h) *HNL1*, (i) *NIT4A*, (j) *NIT4B1*, (k) *NIT4B2*, (l) *CAS1*, (m) *GSTL1*, (n) *GSTL2*. *Y*-axis, transcript level relative to sorghum *PP2A* expression. *X*-axis, developmental stages. S, seedling stage; V, vegetative stage; R, ripening stage. Values are shown as means. Error bars indicate standard deviation. $n = 3$ or more.

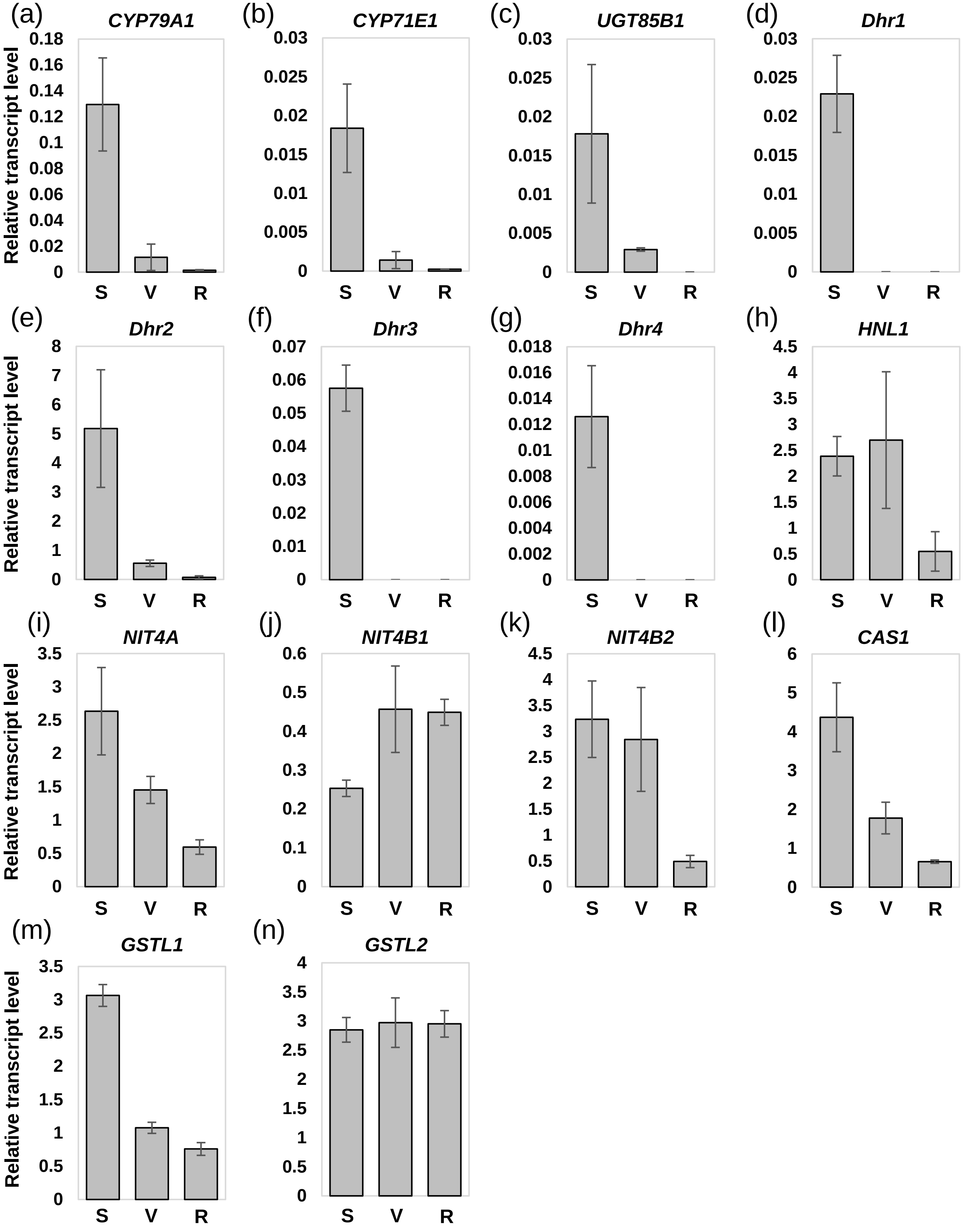

Figure S17. Expression patterns of candidate genes for dhurrin metabolism according to different developmental stages in SAP-265. qRT-PCR analyses of (a) *CYP79A1*, (b) *CYP71E1*, (c) *UGT85B1*, (d) *Dhr1*, (e) *Dhr2*, (f) *Dhr3*, (g) *Dhr4*, (h) *HNL1*, (i) *NIT4A*, (j) *NIT4B1*, (k) *NIT4B2*, (l) *CAS1*, (m) *GSTL1*, (n) *GSTL2*. *Y*-axis, transcript level relative to sorghum *PP2A* expression. *X*-axis, developmental stages. S, seedling stage; V, vegetative stage; R, ripening stage. Values are shown as means. Error bars indicate standard deviation. $n = 3$ or more.

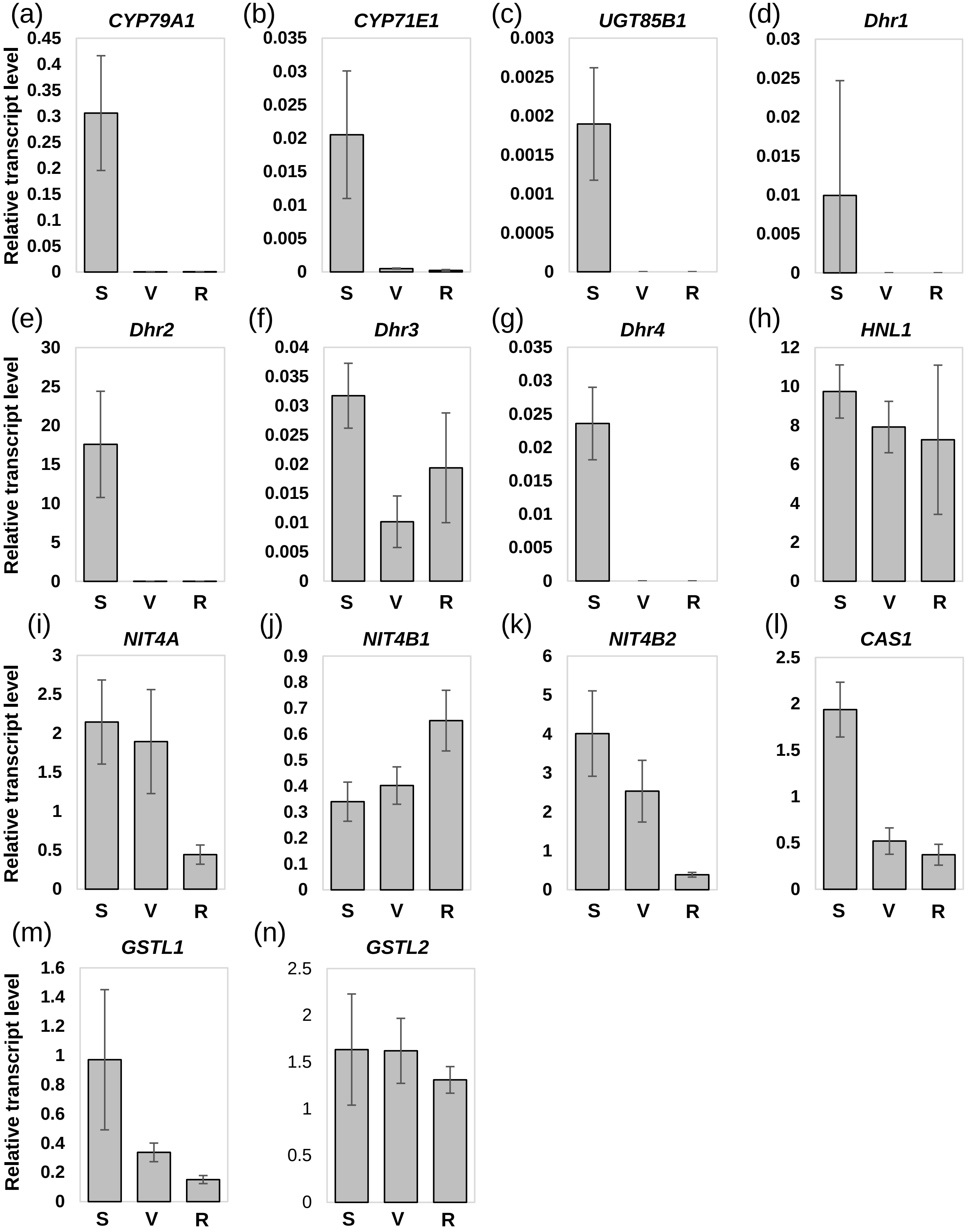

Figure S18. Expression patterns of candidate genes for dhurrin metabolism according to different developmental stages in SAP-272. qRT-PCR analyses of (a) *CYP79A1*, (b) *CYP71E1*, (c) *UGT85B1*, (d) *Dhr1*, (e) *Dhr2*, (f) *Dhr3*, (g) *Dhr4*, (h) *HNL1*, (i) *NIT4A*, (j) *NIT4B1*, (k) *NIT4B2*, (l) *CAS1*, (m) *GSTL1*, (n) *GSTL2*. *Y*-axis, transcript level relative to sorghum *PP2A* expression. *X*-axis, developmental stages. S, seedling stage; V, vegetative stage; R, ripening stage. Values are shown as means. Error bars indicate standard deviation. *n* = 3 or more.

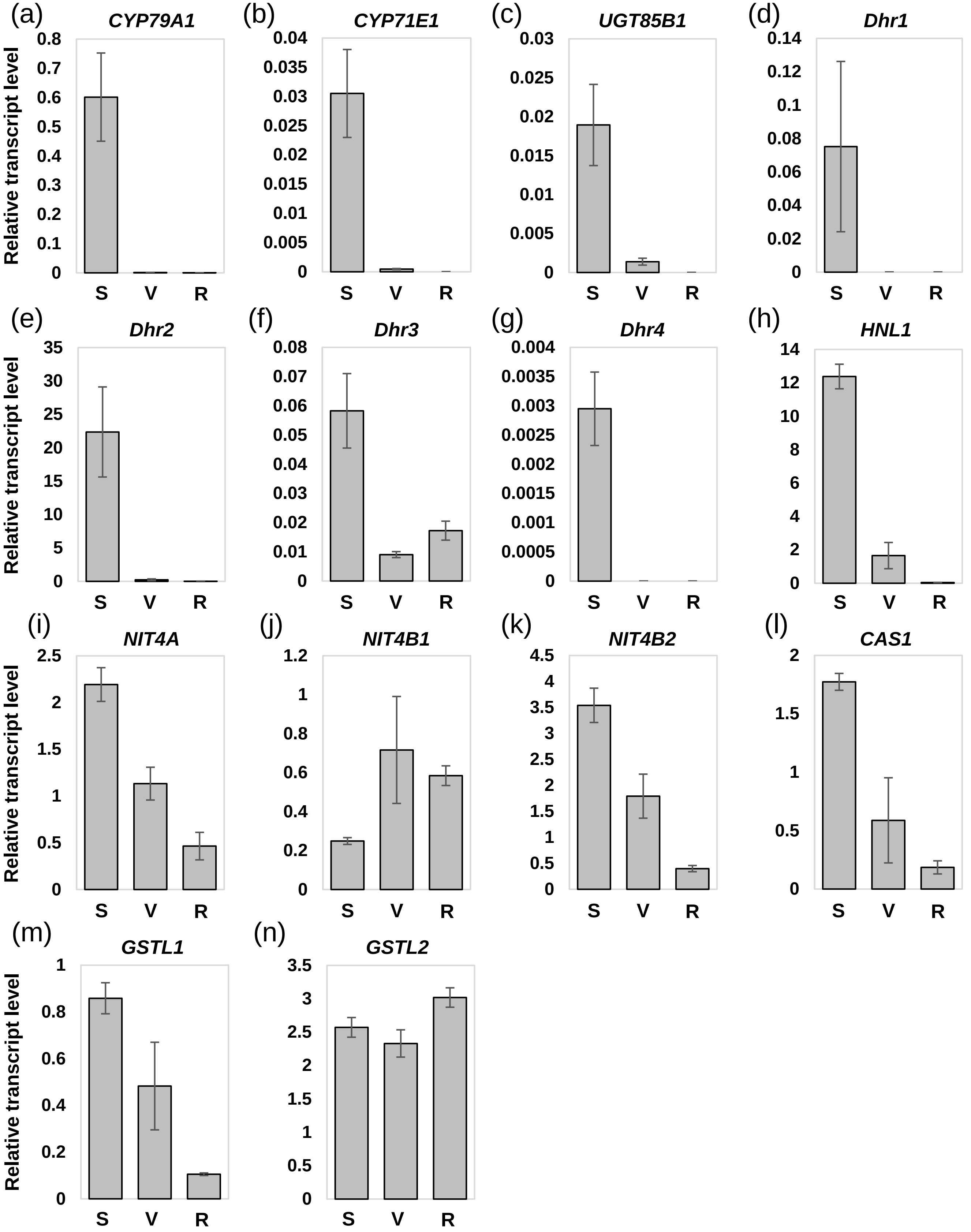

Figure S19. Expression patterns of candidate genes for dhurrin metabolism according to different developmental stages in SAP-317. qRT-PCR analyses of (a) *CYP79A1*, (b) *CYP71E1*, (c) *UGT85B1*, (d) *Dhr1*, (e) *Dhr2*, (f) *Dhr3*, (g) *Dhr4*, (h) *HNL1*, (i) *NIT4A*, (j) *NIT4B1*, (k) *NIT4B2*, (l) *CAS1*, (m) *GSTL1*, (n) *GSTL2*. *Y*-axis, transcript level relative to sorghum *PP2A* expression. *X*-axis, developmental stages. S, seedling stage; V, vegetative stage; R, ripening stage. Values are shown as means. Error bars indicate standard deviation. *n* = 3 or more.

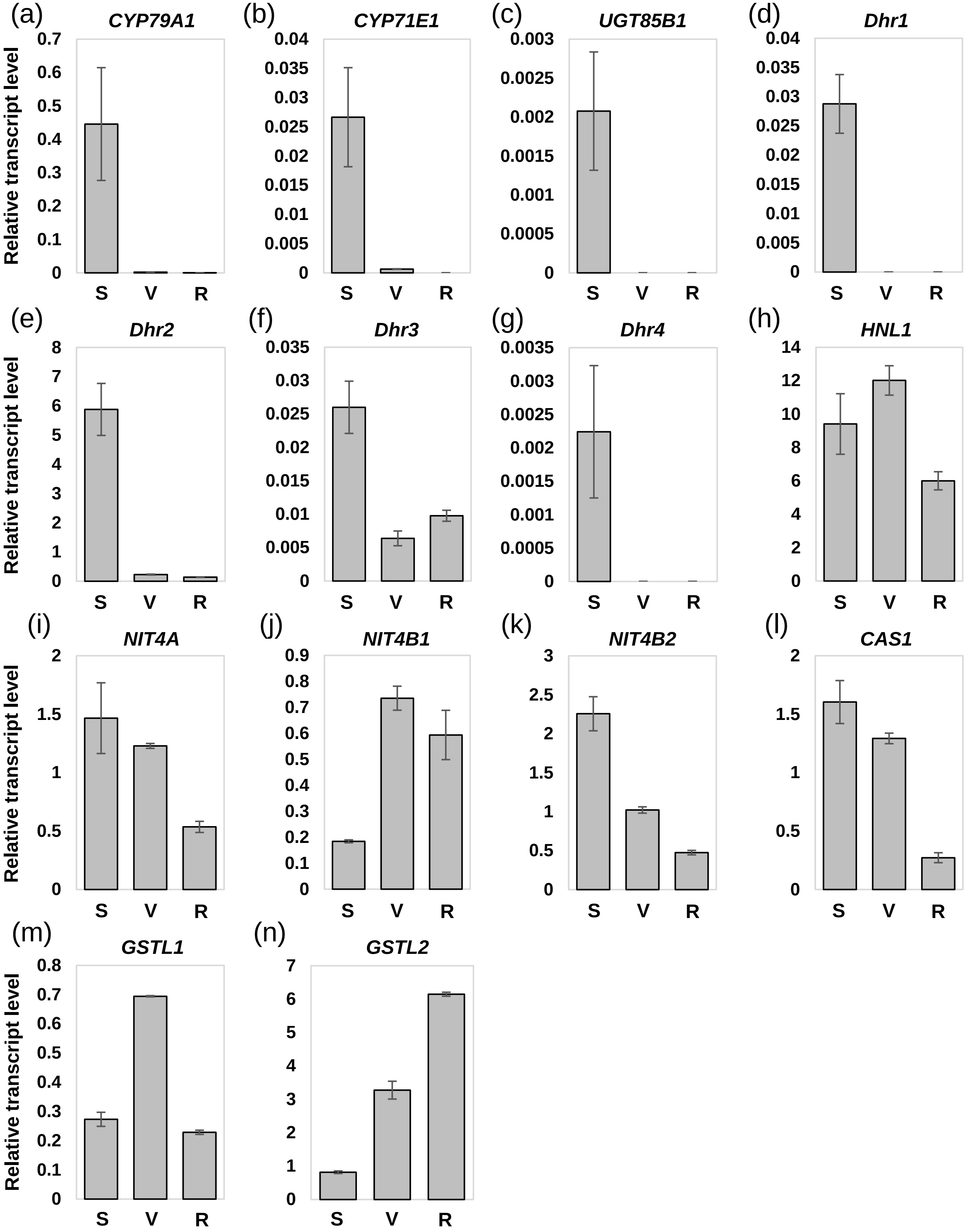

Figure S20. Expression patterns of candidate genes for dhurrin metabolism according to different developmental stages in SAP-336. qRT-PCR analyses of (a) *CYP79A1*, (b) *CYP71E1*, (c) *UGT85B1*, (d) *Dhr1*, (e) *Dhr2*, (f) *Dhr3*, (g) *Dhr4*, (h) *HNL1*, (i) *NIT4A*, (j) *NIT4B1*, (k) *NIT4B2*, (l) *CAS1*, (m) *GSTL1*, (n) *GSTL2*. *Y*-axis, transcript level relative to sorghum *PP2A* expression. *X*-axis, developmental stages. S, seedling stage; V, vegetative stage; R, ripening stage. Values are shown as means. Error bars indicate standard deviation. $n = 3$ or more.

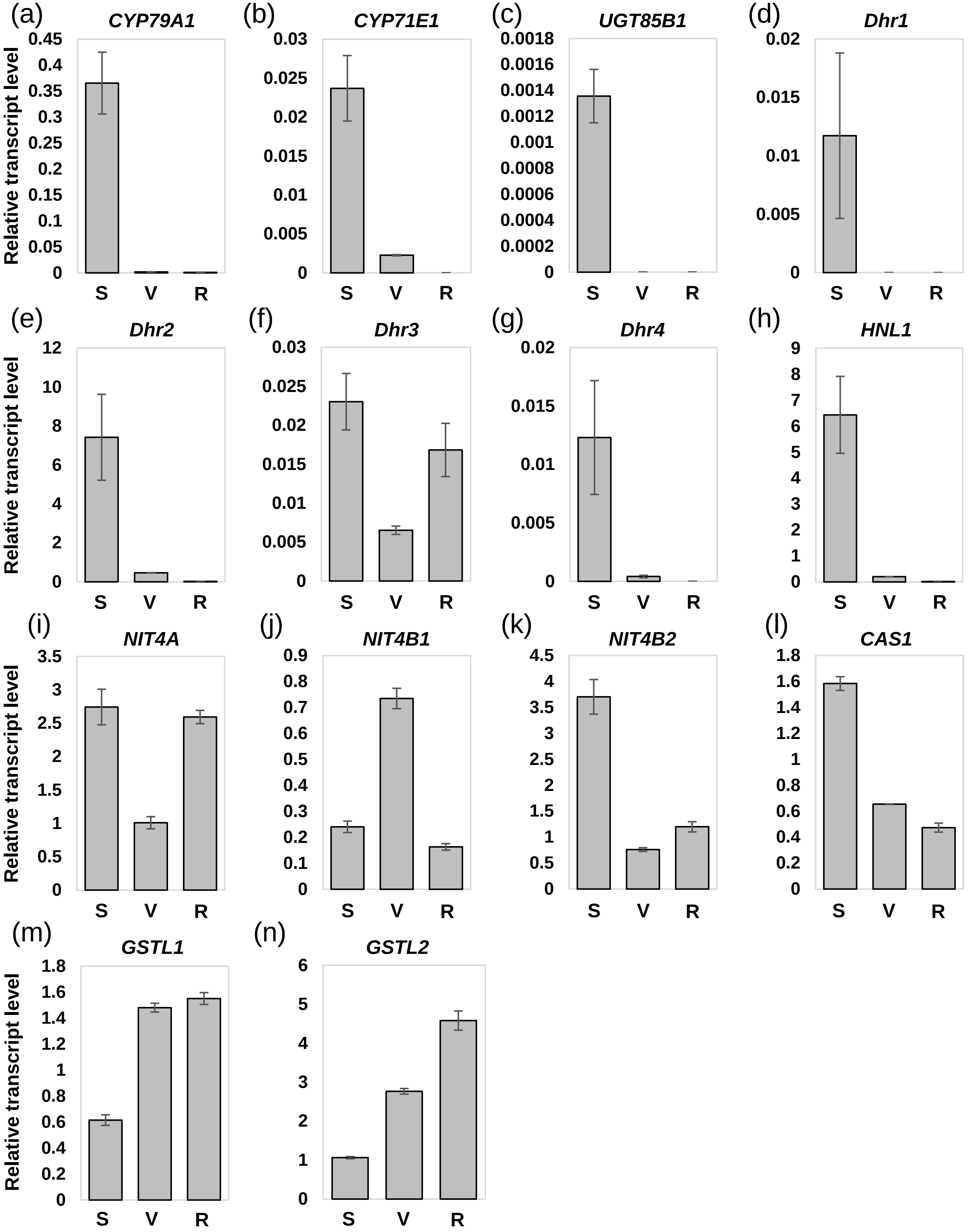

Figure S21. Expression patterns of candidate genes for dhurrin metabolism according to different developmental stages in SAP-354. qRT-PCR analyses of (a) *CYP79A1*, (b) *CYP71E1*, (c) *UGT85B1*, (d) *Dhr1*, (e) *Dhr2*, (f) *Dhr3*, (g) *Dhr4*, (h) *HNL1*, (i) *NIT4A*, (j) *NIT4B1*, (k) *NIT4B2*, (l) *CAS1*, (m) *GSTL1*, (n) *GSTL2*. *Y*-axis, transcript level relative to sorghum *PP2A* expression. *X*-axis, developmental stages. S, seedling stage; V, vegetative stage; R, ripening stage. Values are shown as means. Error bars indicate standard deviation. n = 3 or more.

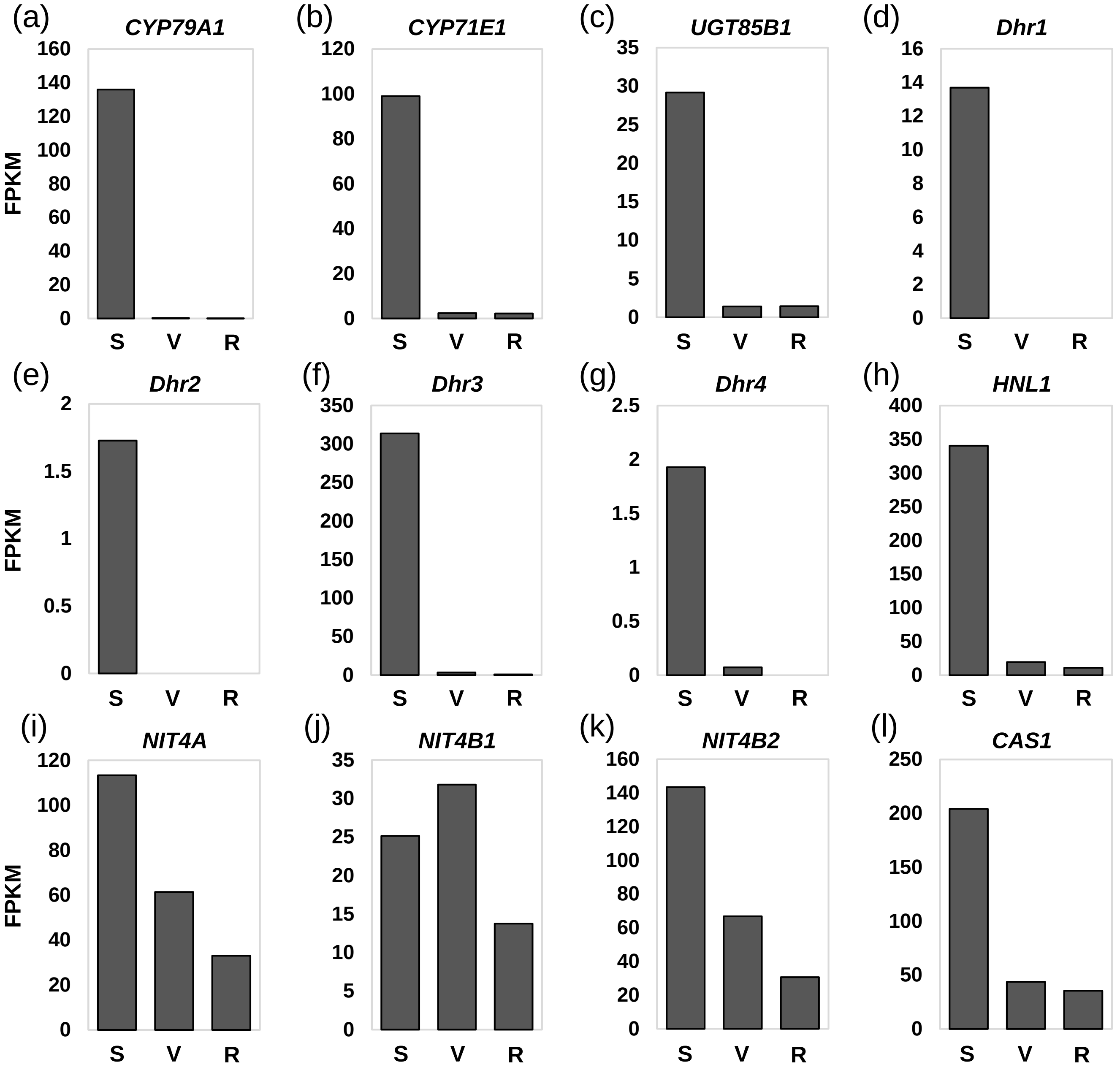

Figure S22. Expression profiles of candidate genes related to dhurrin metabolism in BTx623 using RNAseq data. FPKM analyses of (a) *CYP79A1*, (b) *CYP71E1*, (c) *UGT85B1*, (d) *Dhr1*, (e) *Dhr2*, (f) *Dhr3*, (g) *Dhr4*, (h) *HNL1*, (i) *NIT4A*, (j) *NIT4B1*, (k) *NIT4B2*, (l) *CAS1*. *Y*-axis, FPKM value. *X*-axis, developmental stage. FPKM, fragments per kilobase of exon per million reads mapped; S, seedling stage; V, vegetative stage; R, ripening stage.

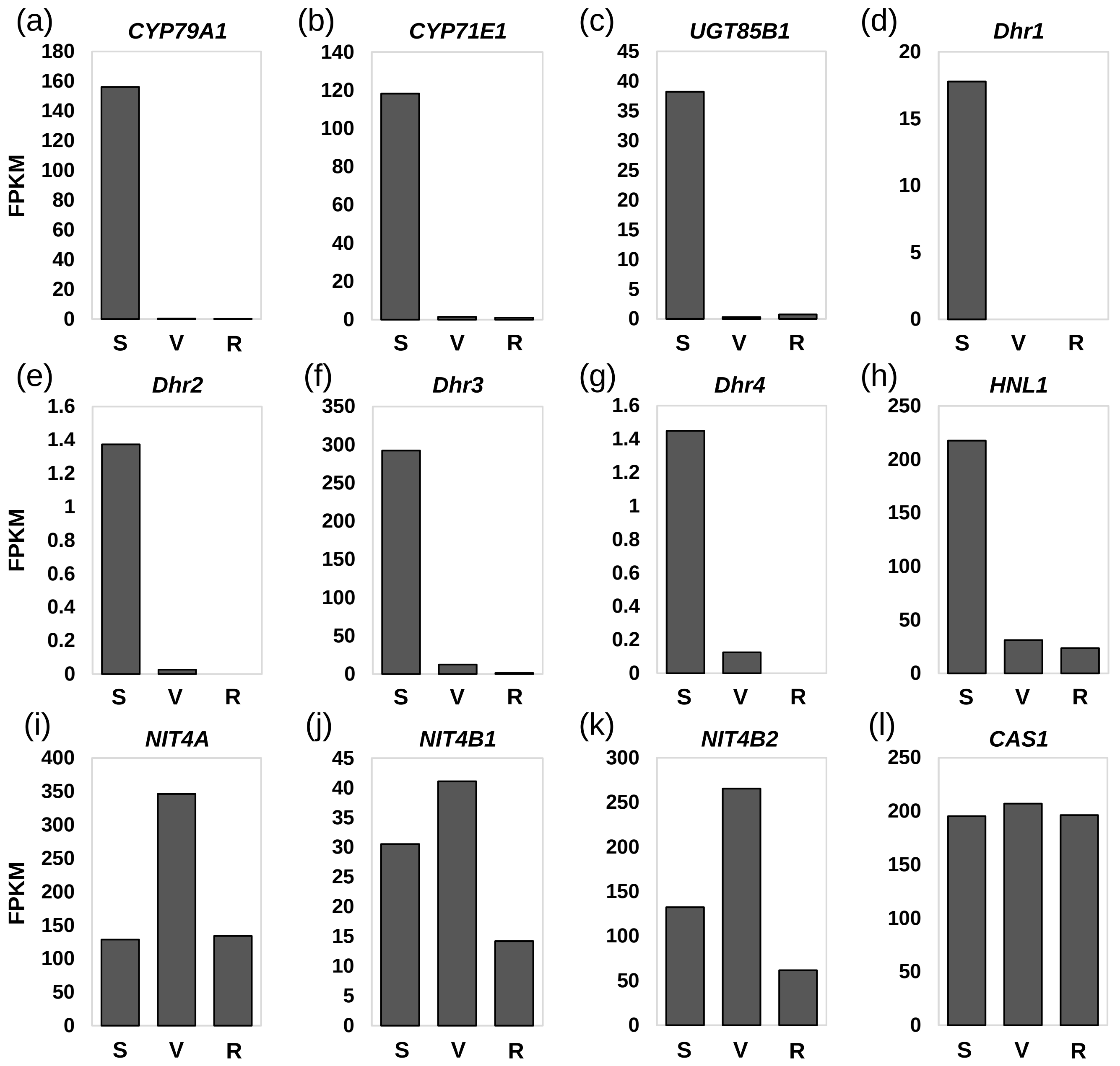

Figure S23. Expression profiles of candidate genes related to dhurrin metabolism in *S. halepense* using RNAseq data. FPKM analyses of (a) *CYP79A1*, (b) *CYP71E1*, (c) *UGT85B1*, (d) *Dhr1*, (e) *Dhr2*, (f) *Dhr3*, (g) *Dhr4*, (h) *HNL1*, (i) *NIT4A*, (j) *NIT4B1*, (k) *NIT4B2*, (l) *CAS1*. *Y*-axis, FPKM value. *X*-axis, developmental stage. FPKM, fragments per kilobase of exon per million reads mapped; S, seedling stage; V, vegetative stage; R, ripening stage.

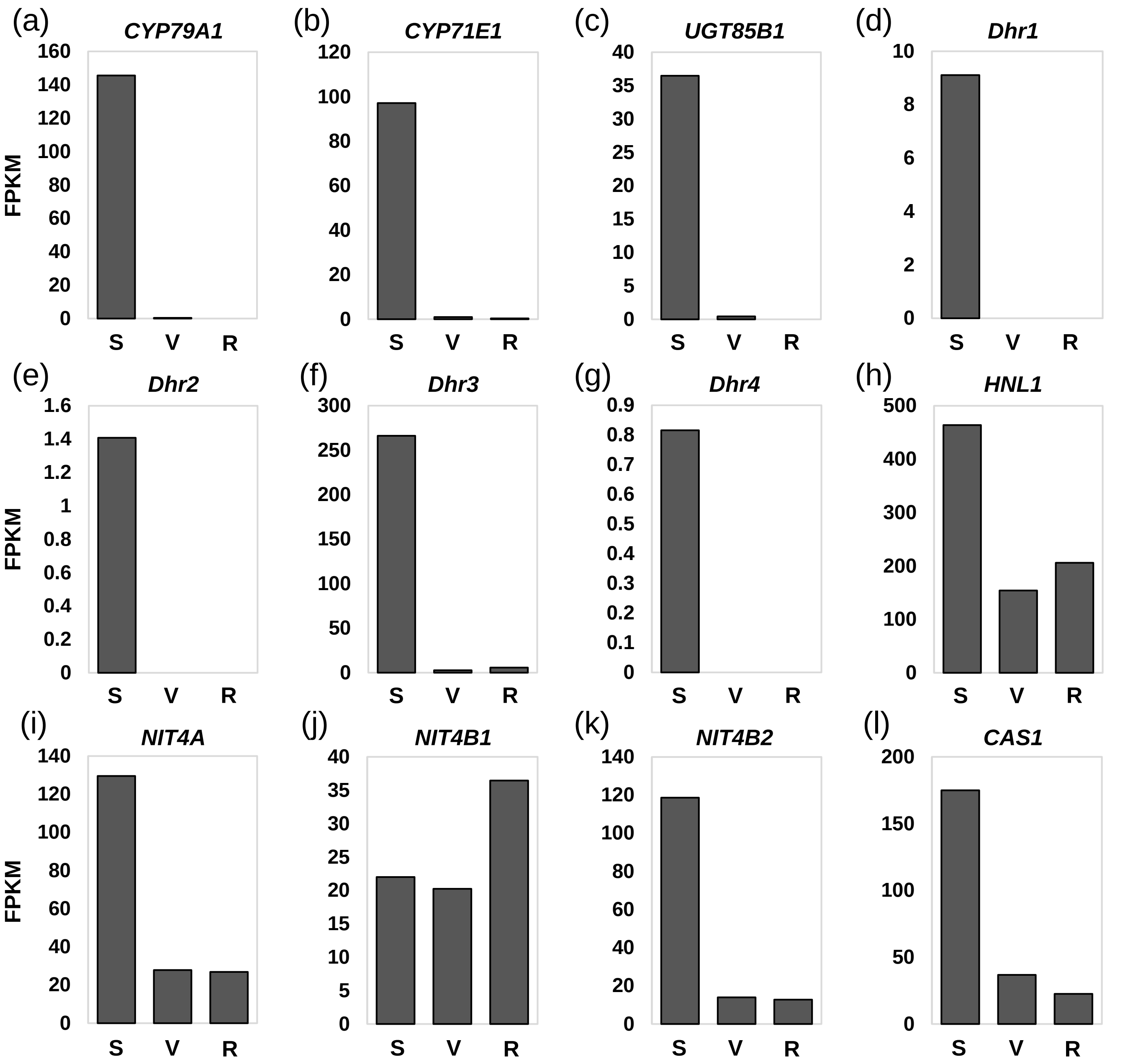

Figure S24. Expression profiles of candidate genes related to dhurrin metabolism in SAP-336 using RNAseq data. FPKM analyses of (a) *CYP79A1*, (b) *CYP71E1*, (c) *UGT85B1*, (d) *Dhr1*, (e) *Dhr2*, (f) *Dhr3*, (g) *Dhr4*, (h) *HNL1*, (i) *NIT4A*, (j) *NIT4B1*, (k) *NIT4B2*, (l) *CAS1*. *Y*-axis, FPKM value. *X*-axis, developmental stage. FPKM, fragments per kilobase of exon per million reads mapped; S, seedling stage; V, vegetative stage; R, ripening stage.

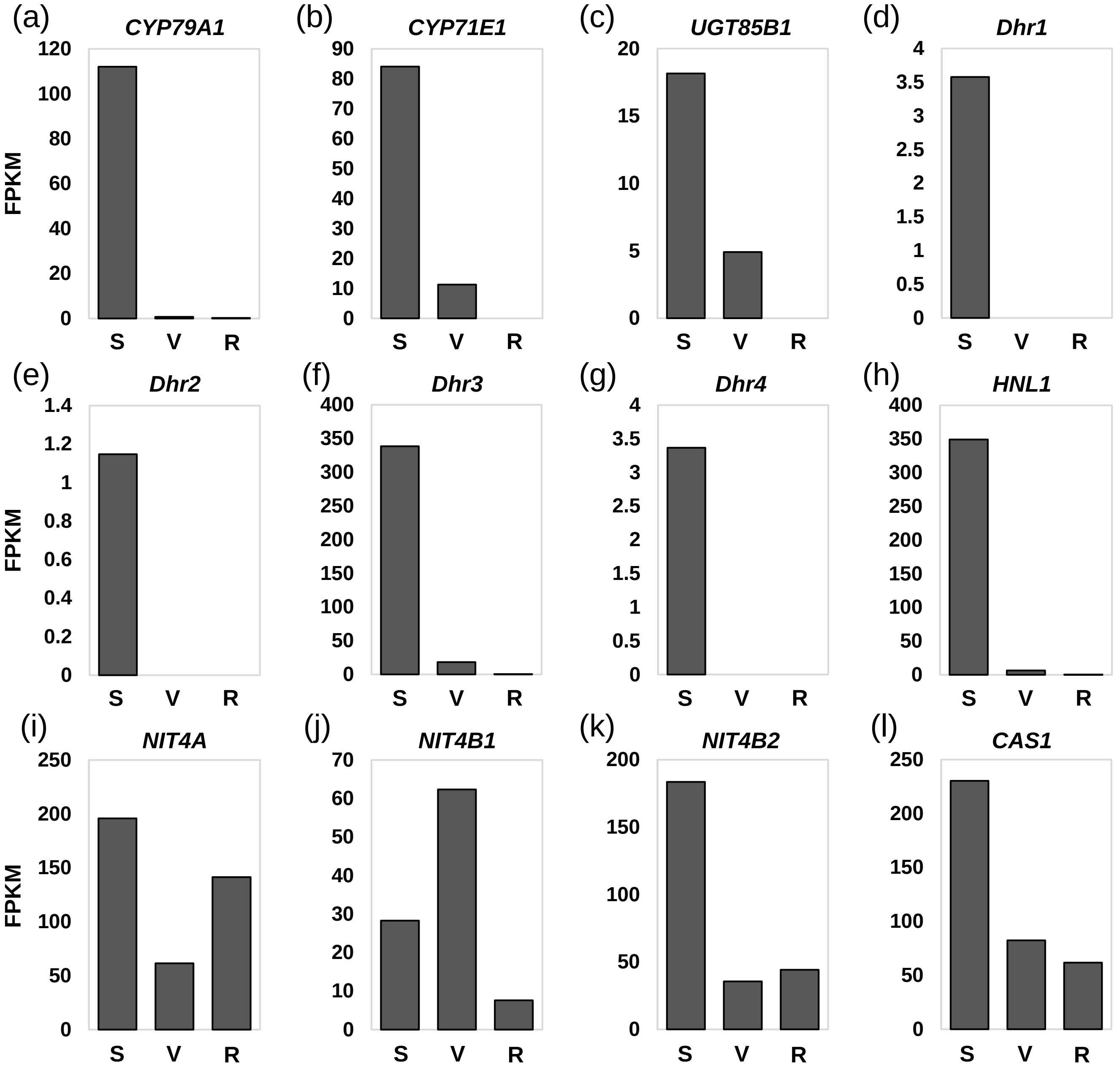

Figure S25. Expression profiles of candidate genes related to dhurrin metabolism in SAP-354 using RNAseq data. FPKM analyses of (a) *CYP79A1*, (b) *CYP71E1*, (c) *UGT85B1*, (d) *Dhr1*, (e) *Dhr2*, (f) *Dhr3*, (g) *Dhr4*, (h) *HNL1*, (i) *NIT4A*, (j) *NIT4B1*, (k) *NIT4B2*, (l) *CAS1*. *Y*-axis, FPKM value. *X*-axis, developmental stage. FPKM, fragments per kilobase of exon per million reads mapped; S, seedling stage; V, vegetative stage; R, ripening stage.

(a)

BTx623

AACACACTGGTGTGGTGAGTGGTGAGTGGCCCGCCTGTCGAGTCTCCTCTCCTCTTCCGTCCTCCACCACCCACC
AATCATCATCACCACCAAAGCTGAGCTGAGCTGAGCTCTCCTGAGTCCCTCACTACTAGCTAAGCTAAGCTAAG
CTAAGCTAGTAGCTCGA**ATG**GAGAGGATGCTGGCAAGGCTGATGCGGCGGCGGAGCTCCTCCCCCCTGTCCGACC
TCCTCCACCATGGAGGAGCAGCAGCGGCGGGCTCGCTGCAGGCCGCCGCCGGCGCCACTGCGGCCTCCCCCTGGC
TCTTCTCCCACCACCAGCAGCAGCAGCAGCAGCACACGGCGGCCGCCGCGCTGCCGGGTCTCAAGATCAGGGAC
TCCGCGTCCCAGCTGATTGGGAGGACACCGATGGTGTACCTGAACAAGGTGACGGAGGGATGCGGCGCCCGGAT

S. halepense

AACACACTGGTGTGGTGAGTGGTGAGTGGCCCGCCTGTCGAGTCTCCTCTCCTCTTCCGTCCTCCACCACCCACC
AATCATCATCAGCAGTGAGCTGAGCTCTCCTGAGTCCCTCACTACTAGCTAAGCTAAGCTAATAGCTCGA**ATG**G
AGAGGATGCTGGCAAGGCTGATGCGGCGGCGGAGCTCCTCCCCCCTGTCCGACCTCCTCCACCATGGAGGAGCAG
CAGCGGCGACGGGCTCGCTGCAGGCCGCCGCCGGCGCCACTGCGGCCTCCCCCTGGCTCTTCTCCCACCACCAC
ACCAGCAGCAGCACACGGCGGCCGCCACGCTGCCGGGTCTCAAGATCAGGGACTCCGCGTCCCAGCTGATTGGT
AGGACACCGATGGTGTACCTGAACAAGGTGACGGAGGGATGCGGCGCCCGGATCGCTGCCAAGCTCGAGTTCCTG

SAP-336

AACACACTGGTGTGGTGAGTGGTGAGTGGCCCGCCTGTCGAGTCTCCTCTCCTCTTCCGTCCTCCACCACCCACC
AATCATCATCATCCACCAAAGCTGAGCTGAGCTGAGCTCTCCTGAGTCCCTCACTACTAGCTAAGCTAAGCTAAG
CTAATAGCTCGA**ATG**GAGAGGATGCTGGCAAGGCTGATGCGGCGGCGGAGCTCCTCCCCCCTGTCCGACCTCCTC

(b)

BTx623

MERMLARLMRRRSSSPLSDLLHHGGAAAAGSLQAAAGATAASPWLFSHHQQQQQHTAAAALPGLKIRDSASQLI
GRTPMVYLNKVTEGCGARIAAKLEFLQPSFSVKDRPAISMLEDAEKRGLITPGKTTLIEPTSGNMGIGLAFMAAL
KGYELILTMPSYTSLERRVTMRAFGANLVLTDPTKGMGGTVRKAAELYEKHPSAYMLQQFQNPANVKVHYETTGP
EIWEDTLGQVDIFVMGIGSGGTVTGVGKYLKEKNPNAKIYGVEPAEANVLNGGKPGPHLITGNGVGFKPDILDMD
VMEKVLEVKGEDAVKMARELALKEGLLVGISSGANTVAALELAKKPENKGKLIVTVLPSLGERYLSSALFDELRK
EAEAMEPVPVD*

S. halepense

MERMLARLMRRRSSSPLSDLLHHGGAAAATGSLQAAAGATAASPWLFSHHHTSSSTRRPPRCRVSRSGTPRPS*

Figure S26. Raw sequence of *CAS1* by SNP analysis. (a) Nucleotide sequences of *CAS1* in BTx623, *S. halepense*, and SAP-336 (b). Deduced amino acid sequences of CAS1 in BTx623 and *S. halepense*. Green shading represents 5' UTR and light blue shadings represent exons. Blue letters with black border indicate positions of SNPs in accession BTx623, and red letters with black border indicate counterparts to each SNP position on BTx623 sequence.
